# We are the champions. The index for evaluating concentration of championships using a sliding window approach

**DOI:** 10.1016/j.heliyon.2022.e12025

**Published:** 2022-12-05

**Authors:** Ivan Simko

**Affiliations:** U.S. Department of Agriculture, Agricultural Research Service, U.S. Agricultural Research Station, Crop Improvement and Protection Research Unit, 1636 E. Alisal Street, Salinas, CA, 93905, USA

**Keywords:** Individual sport, Team sport, Grouping of champions, Sliding windows, Resampling, Trends

## Abstract

The concept of competitive balance is considered to be an essential aspect in the field of sport economics. This work describes a novel approach for measuring and evaluating competitive balance through concentration of championships. The concentration of championships was assessed using a sliding window approach with the length of five consecutive competitions (years) and a single competition (year) increment over the whole evaluated period. Because the sliding window was relatively short, the newly developed index, termed ‘We Are the Champions’ (WAC_5_), is sensitive to rapid changes in competitive balance. The WAC_5_ index, average WAC_5_ (aWAC_5_), expected WAC_5_ (eWAC_5_), and ΔWAC_5_ (eWAC_5_ - a_WAC_5__) were applied to data from 68 competitions of several individual and team sports collected for the 1960–2020 period. The significance of indices was tested by resampling (bootstrapping and permutation). The results of the study show a growth in competitive balance of several ice hockey competitions (national and international), but decreasing competitive balance in Formula 1 racing and in several European soccer competitions. In soccer competitions, there was a substantially lower competitive balance in a league than in a domestic cup competition within each country/federation. The difference between the overall competitive balance in the most popular, North American, professional leagues and the top European soccer leagues is growing. A significant grouping of champions was determined for all sports involving individual athletes, but also for several team competitions.

## 1 Introduction

“We are the champions, my friends, and we'll keep on fighting 'til the end” sings Freddie Mercury from the British rock band Queen [1] in ‘We are the Champions’, a song that is popular among fans and athletes celebrating sport victories. Though only relatively few athletes can actually become champions, the prospect of winning a championship creates euphoria among athletes and fans alike. Participation in sport and demand for sport-related goods, live events, and broadcast have all risen steeply in North America since 1970s, Western Europe since 1980s, and in most of the other areas of the world since 1990s [2]. The estimated size of the global sports industry in 2018 was $1.3 trillion [3]. Based on the global financial sport market, the number one sport is soccer (association football) with 43% market share, followed by American football (13%), baseball (12%), Formula 1 racing (7%), basketball (6%), ice hockey (4%), tennis (4%), and golf (3%) [4]. The relatively unpredictable nature of sport with an uncertainty of outcome adds to the overall excitement for fans and may affect attendance of sport events [5]. Therefore, fans, managers, athletes, and also some economists occasionally express concern about the growing concentration of contenders to win championships [6, 7]. To improve competitive balance among teams or individual athletes, several sport governing bodies made changes in a league or a tournament format and developed policies such as salary limitations, revenue sharing, draft rules, or roster limits [6, 8]. However, the lack of competitive balance in major European soccer leagues where the majority of titles have been won by only a few teams, together with extraordinary popularity of these leagues indicates that fans may be less concerned with competitive balance than might be expected [2, 8, 9].

Competitive balance can be measured as a within-season or between-seasons variation. The first evaluates dispersion of wins, points, or other parameters among teams in a specific season, while the second one analyses results across several seasons or tournaments. There are many methods to measure these variations, and no single method should be regarded as most appropriate [8]. The within-season, competitive balance has been evaluated for a number of individual or team sports, including baseball, basketball, football, ice hockey, soccer, speed skating, table tennis, and tennis using the Herfindahl-Hirschman Index (HHI), Competitive Balance Ratio (CBR), the ratio of standard deviations (RSD), Gini coefficient and related Lorenz curve, relative entropy, and other, specific-sport-based criteria, such as points, scores, or time [10, 11, 12, 13, 14, 15, 16, 17, 18, 19, 20, 21, 22]. Between-seasons variation can be evaluated for an individual team using the team-specific variation in standing in different seasons (turnover) [11], or by measuring the concentration of championships in a given time period [8]. Because indices measuring the concentration of championships are based only on a relative frequency of championship success, they are unaffected by the distribution of wins, points, or scores accumulated during championships or by the championships format. This feature allows comparing indices of competitive balance across multiple sports and eras.

Previous studies analyzed concentration of championships across only a few competitions within a certain time period [8, 23, 24], or for a single competition (occasionally more) in a few, non-overlapping time periods [23, 25]. Indices calculated from a whole evaluating period, however, do not allow for a detection of gradual changes in concentration of championships. The present study describes the use of a sliding window approach (together with novel indices) to detect gradual changes in concentration of championships. Because values in subsequent sliding windows are not independent from each other, statistical analyses of indices were performed using the model-free, block bootstrapping approach.

The concentrations of championships were evaluated in more than 60 major, professional leagues and tournaments, amateur (college level) tournaments, and some less economically influential leagues and competitions using a novel approach based on resampling. Though most studies in the literature analyze competitive balance in team sports, rather than in individual sports [13], this study includes for comparison also several tournaments for individual athletes. The objectives of the study were to use the newly developed indices and resampling approach to (1) compare differences in concentrations of championships across competitions, (2) evaluate trends in concentration of championships, and (3) determine a relative grouping of champions.

## 2 Data collection

Data were collected for several popular sport leagues and major tournaments and also for some lesser known or amateur championships. These sports include association football (‘soccer’ henceforth), American football and Australian rules football (both ‘football’ henceforth), basketball, baseball, curling, ice hockey, and rugby union (‘rugby’ henceforth), and from individual sports tennis, golf, and Formula 1 racing (‘F1’ henceforth) (Table 1). Links to webpages with listed champions are provided in Online Resource 1. The effort has been made to keep only a single name for a team that modified its original name but was still considered to be an official successor of the original team. In women's individual sports, maiden names were matched to married names. When a league or a tournament was not held in some years, or a champion has not been declared, these years were omitted from data analysis. In four exceptions, two teams were declared to be official champions. In these situations, the calculations were performed using each champion separately and then averaging results. Though data were collected for all documented champions in evaluated competitions (some of them starting at the end of 19^th^ century), only the period from 1960 to 2020 was considered for the statistical analyses. This period approximately matches with the historical growth in popularity of sport [2] and also has more complete data for a larger number of leagues and tournaments than the older time period.

**Table 1:** List of 68 analyzed competitions.

Competition^a^	Sport	Details	Country or region	Category	Classification	Years
B-NBA	Basketball		USA/Canada	Men	Team	1947–2019
B-NCAA	Basketball	College	USA	Men	Team	1939–2019
B-NCAA-W	Basketball	College	USA	Women	Team	1982–2019
B-Spa	Basketball		Spain	Men	Team	1958–2019
Ba-Jap	Baseball		Japan	Men	Team	1950–2019
Ba-Mex	Baseball		Mexico	Men	Team	1946–2020
Ba-MLB	Baseball		USA/Canada	Men	Team	1903–2019
C-WC	Curling		International	Men	Team	1959–2019
C-WC-W	Curling		International	Women	Team	1979–2019
F-AFL	Football-AU		Australia	Men	Team	1897–2019
F-NFL	Football-US		USA	Men	Team	1967–2020
F1	Formula 1		International	-	Individual	1950–2019
F1-E^a^	Formula 1	Engine	International	-	-	1950–2019
G-Mas	Golf		International	Men	Individual	1934–2019
G-Open	Golf		International	Men	Individual	1870–2019
G-PGA	Golf		International	Men	Individual	1916–2019
G-US	Golf		International	Men	Individual	1895–2019
IH-NHL	Ice hockey		USA/Canada	Men	Team	1915–2019
IH-Rus	Ice hockey		Russia	Men	Team	1947–2019
IH-Svk	Ice hockey		Slovakia	Men	Team	1994–2019
IH-Swe	Ice hockey		Sweden	Men	Team	1922–2019
IH-WC	Ice hockey		International	Men	Team	1920–2019
IH-WC-W	Ice hockey		International	Women	Team	1990–2019
R-Eng	Rugby		England	Men	Team	1988–2019
R-Fra	Rugby		France	Men	Team	1892–2019
R–I&NI	Rugby		Ireland/North Ireland	Men	Team	1991–2019
R-Ken	Rugby		Kenya	Men	Team	1970–2019
S-Bel	Soccer	League	Belgium	Men	Team	1896–2019
S-Bel-C	Soccer	Cup	Belgium	Men	Team	1912–2019
S-Bra	Soccer		Brazil	Men	Team	1959–2019
S-CL	Soccer		International	Men	Team	1956–2019
S-CL-C^a^	Soccer	Countries	International	Men	-	1956–2019
S-CSR	Soccer	League	Czechoslovakia	Men	Team	1946–1993
S-CSR	Soccer	Cup	Czechoslovakia	Men	Team	1961–1993
S-CWC	Soccer		International	Men	Team	1961–1999
S-CWC-C^a^	Soccer	Countries	International	Men	Team	1961–1999
S-Cze	Soccer	League	Czechia	Men	Team	1895–2019
S-Cze-C	Soccer	Cup	Czechia	Men	Team	1961–2019
S-EL	Soccer		International	Men	Team	1972–2019
S-EL-C^a^	Soccer	Countries	International	Men	Team	1972–2019
S-Eng	Soccer	League	England/Wales	Men	Team	1889–2019
S-Eng-C	Soccer	Cup	England	Men	Team	1972–2019
S-Fra	Soccer	League	France	Men	Team	1894–2019
S-Fra-C	Soccer	Cup	France	Men	Team	1918–2019
S-Ger	Soccer	League	Germany	Men	Team	1903–2019
S-Ger-C	Soccer	Cup	Germany	Men	Team	1935–2019
S-Ita	Soccer	League	Italy	Men	Team	1898–2019
S-Ita-C	Soccer	Cup	Italy	Men	Team	1922–2019
S-MLS	Soccer		USA/Canada	Men	Team	1996–2019
S-NCAA	Soccer	College	USA	Men	Team	1959–2019
S-Net	Soccer	League	The Netherlands	Men	Team	1889–2019
S-Net-C	Soccer	Cup	The Netherlands	Men	Team	1899–2019
S-Por	Soccer	League	Portugal	Men	Team	1935–2019
S-Por-C	Soccer	Cup	Portugal	Men	Team	1922–2019
S-Sco	Soccer	League	Scotland	Men	Team	1891–2019
S-Sco-C	Soccer	Cup	Scotland	Men	Team	1874–2019
S-Spa	Soccer	League	Spain	Men	Team	1929–2019
S-Spa-C	Soccer	Cup	Spain	Men	Team	1903–2019
S-Svk	Soccer	League	Slovakia	Men	Team	1925–2019
S-Svk-C	Soccer	Cup	Slovakia	Men	Team	1961–2019
T-Aus	Tennis		International	Men	Individual	1905–2020
T-Aus-W	Tennis		International	Women	Individual	1922–2020
T-Eng	Tennis		International	Men	Individual	1877–2019
T-Eng-W	Tennis		International	Women	Individual	1884–2019
T-Fra	Tennis		International	Men	Individual	1891–2019
T-Fra-W	Tennis		International	Women	Individual	1897–2019
T-USA	Tennis		International	Men	Individual	1881–2019
T-USA-W	Tennis		International	Women	Individual	1887–2019

Links to original data and additional notes about competitions are provided in Online Resource 1.

a Indicates four competitions that are not official but were added to the list to evaluate additional trends.

## 3 Measuring competitive balance

This study uses a sliding window approach to evaluate distribution of champions in five consecutive years, with a single year increment. Such a relatively short period of time has been selected to better observe rapid changes in trends. In addition, when the number of competitions in the evaluated period is smaller than the number of teams or athletes participating in these individual competitions, no adjustment is needed to account for the number of participating teams when calculating the maximum competitive balance [8].

Several approaches can be used to evaluate concentration of championships from the fixed number of competitions that is not larger than the number of competing teams. To calculate the average concentration of championships at any given, five-year period, this study uses the average difference in the number of championships won by individual teams (For simplicity, the term ‘team’ is used in descriptions, but the same approach applies to competitions of individual athletes. Similarly, terms ‘years’, ‘championships’, ‘tournaments’, and ‘competitions’ may be used interchangeably when describing the length of the evaluated period). Five consecutive championships (with a single champion in each of them) could be won by up to five teams. If one or more teams win multiple championships, the number of unique champions decreases. Therefore, the maximum competitive balance is reached when each championship is won by a different team. These different champions can be written as ABCDE (or A = 1, B = 1, C = 1, D = 1, and E = 1), and the average difference between wins of these five teams equals 0. Opposite, the minimum competitive balance is reached when a single team wins all five championships; AAAAA (or A = 5, B = 0, C = 0, D = 0, and E = 0). In this case, the average difference in the number of wins between 10 pairs of these five teams would be 20/10 = 2. The average absolute differences for the other five, possible combinations of champions could be similarly calculated; 1.8 for AAAAB, 1.6 for AAABB, 1.4 for AAABC, 1.2 for AABBC, and 0.8 for AABCD. These values then can be scaled to the 0.0 (minimal competitive balance) to 1.0 (maximal competitive balance) range using formula: ‘scaled value’ = (‘original value’—2)/(– 2). This scaled value has been termed ‘WAC_5_’ (We Are the Champions, 5-year) index. The WAC_5_ values for the seven possible combinations of champions are: AAAAA = 0.0, AAAAB = 0.1, AAABB = 0.2, AAABC = 0.3, AABBC = 0.4, AABCD = 0.6, and ABCDE = 1.0. The WAC_5_ index thus takes into the consideration not only the number of unique champions during the five-year period, but also the distribution of wins among these champions (i.e., difference between AAAAB = 0.1 and AAABB = 0.2, and between AAABC = 0.3 and AABBC = 0.4). Moreover, this index gives a larger weight to the last step leading to the maximum competitive balance (from AABCD = 0.6 to ABCDE = 1.0) than to the first step from the minimum competitive balance (from AAAAA = 0.0 to AAAAB = 0.1). Such increased weight has been selected intentionally to accentuate the maximum competitive balance, however, different weights (or indices) could be used with the sliding window approach, if preferred.

## 4 WAC_5_ comparison to other indices

Besides WAC_5_, several other indices could be used to measure competitive balance through the concentration of championships. Because the maximum number of potential champions within a five-year period is exactly five (*n* = 5), the indices based on only these five potential champions are invariant to the total number of teams in the evaluated league (or to the change in the number of participating teams unless the number decreases below five). Notice an important difference among the maximum number of potential champions within a five-year period (*n* = 5), the number of teams that could potentially become champions (all teams participating in those championships), and the actual number of teams that won championships (*N* from 1 to 5). For example, a five-year record from a league with seven teams where four of the teams were champions (*N* = 4) can be written as A = 2, B = 1, C = 1, D = 1, E = 0, F = n.a., and G = n.a, where ‘n.a.’ means not applicable (not used in data analyses because no more than five teams could actually win championships).

When the concentration of championships is evaluated on the fixed number of years (*T* = 5) using the fixed number of potential champions (*n* = 5), several indices can be calculated from the actual number or the proportion of championships (*p*_*i*_) won by each of these five teams. It is important to emphasize, however, that these indices can only be used to estimate a between-season concentration of championships, but they do not indicate the strength of competitive balance within any of the evaluated seasons. To compare performances of indices with a dissimilar range and/or orientation, all indices had needed to be scaled to the 0.0 (for AAAAA) to 1.0 (for ABCDE) range [26] as ‘scaled index value’ = (‘original index value’—‘original index value at AAAAA’)/(‘original index value at ABCDE’—‘original index value at AAAAA’).

### 4.1 Number of champions

Counting the number (*N*) of teams that have won championships, regardless of the number of times each of the teams won, is a probably the simplest way of evaluating concentration of championships. The more teams were the champions, the higher was the competitive balance. The *N* value ranges from 1 (for AAAAA) to 5 (for ABCDE). The scaled value of *N* (*N*_*s*_) is calculated as:

*N*_*s*_ = (*N*—1)/(5–1) = (*N*—1)/4

### 4.2 Variance

When five different teams win one championship, each of them has the winning frequency of *p*_*i*_ = 0.2. If only a single team wins all championships, *p*_*i*_ for this team is 1.0, while for the four other teams that could potentially be winners during this period it is 0.0. The variance (*σ*^*2*^) for the concentration of championships is calculated as:

*σ*^*2*^ = (*∑* (*p*_*i*_—1/*T*)^2^)/(*T*-1) = (*∑* (*p*_*i*_—1/5)^2^)/(5–1) = (*∑* (*p*_*i*_—0.2)^2^)/4

The *σ*^*2*^ values before scaling range from 0.2 (for AAAAA) to 0.0 (for ABCDE). The scaled value of *σ*^*2*^ (*σ*^*2*^_*s*_) is calculated as:

*σ*^*2*^_*s*_ = (*σ*^*2*^—0.2)/(0–0.2) = (*σ*^*2*^—0.2)/(– 0.2)

### 4.3 Standard deviation

Standard deviation (*σ*) is a square root of the variance (*σ*^*2*^), thus the values of an unscaled *σ* range from 0.4472 (for AAAAA) to 0.0 (for ABCDE). The *σ* index is calculated as:

*σ* = √*σ*^2^

The scaled value of *σ* (*σ*_*s*_) is calculated as:

*σ*_*s*_ = (*σ*—0.4472)/(0–0.4472) = (*σ*—0.4472)/(– 0.4472)

### 4.4 Simpson diversity index and its derivatives

The Simpson diversity index [27] used in ecology is equivalent to the Herfindahl-Hirschman index (HHI) for market concentration [28] that was previously applied to evaluate concentration of championships in a given time period [8]. When used for both the fixed number of years and the fixed number of potential champions, the Simpson index is calculated as:

*D* = *∑* (*p*_*i*_)^2^

The values for *D* range from 1.0 (for AAAAA) to 0.2 (for ABCDE). The Simpson diversity index derivative that is also frequently used to evaluate diversity is the inverse (or reciprocal) Simpson index calculated as 1/*D*. Values for 1/*D* at *T* = *n* = 5 range from 1.0 (for AAAAA) to 5.0 (for ABCDE). Another derivative of the Simpson index used in diversity studies is 1-*D*, however this index yields the same scaled values as *D* and therefore it was not considered in the current evaluations.

The scaled value of *D* (*D*_*s*_) is calculated as:

*D*_*s*_ = (*D*—1.0)/(0.2–1.0) = (*D*—1.0)/(– 0.8)

The scaled value of 1/D (1/Ds) is calculated as:

1/*D_s_* = (1/*D*—1.0)/(5.0–1.0) = (1/*D*—1.0)/4

### 4.5 Shannon index

The Shannon index has been originally proposed to quantify entropy in text [29] but is likely the most preferred diversity index in ecology. When applied to evaluate concentration of championships, the index is calculated as:

*H’* = - ∑ *p_i_* ln(*p_i_*)

The index ranges from 0.0 (for AAAAA) to ln(*T*) = 1.6094 (for ABCDE). The scaled values of Shannon index (*H'_s_*) are equivalent to the Pielou evenness (*J′*) index [30].

The scaled value of H’ (H's) is calculated as:

*H'_s_* = (*H’*—0.0)/(1.6094–0.0) = *H’*/1.6094

### 4.6 Gini coefficient

The Gini coefficient [31] that measures variability of distribution is frequently used in economic studies to compare inequality in wealth. When used to compare competitive balance on the fixed number of years and the fixed number of potential champions (*T* = *n* = 5), the coefficient is calculated as:

*G* = (∑ abs (*p_i_*—*p_j_*)) × mean *p* = (∑ abs (*p_i_*—*p_j_*)) × 0.2

Where ‘mean p’ is the average proportion of wins. The unscaled coefficient ranges from 0.8 (for AAAAA) to 0.0 (for ABCDE).

The scaled value of *G* (*G_s_*) is calculated as:

*G_s_* = (*G*—0.8)/(0.0–0.8) = (*G*—0.8)/(- 0.8)

### 4.7 Comparison of scaled indices

When the number of years and the maximum number of potential champions were fixed (*T* = *n* = 5), several indices yielded identical scaled values; the Simpson diversity index (*D*_*s*_) = the Herfindahl-Hirschman index (HHI) = the index based on variance (*σ*^*2*^_*s*_), the Shannon index (*H'*_*s*_) = the Pielou evenness index (*J′*), and the We Are the Champions index (WAC_5_) = the Gini coefficient (*G*_*s*_). In the case of identical results for two or more indices, only results from one of them are presented.

**Figure 1:**
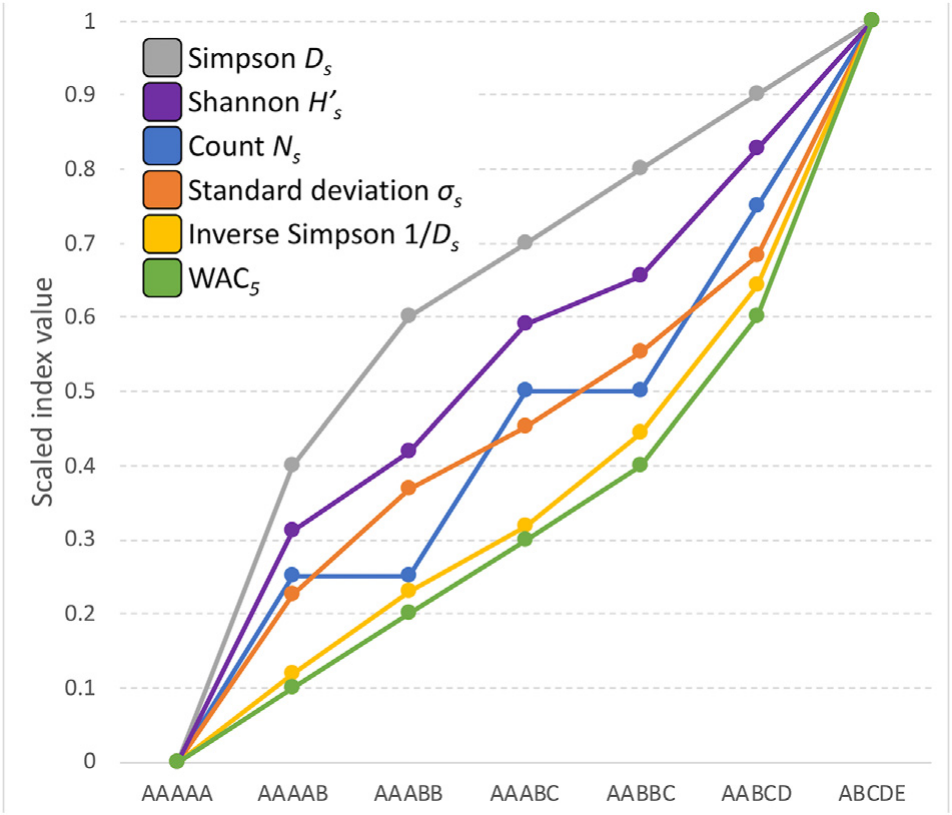
Scaled values of six indices. Letters A to E indicate how many times a team with the corresponding name won the championship in the five-year period.

**Figure 2:**
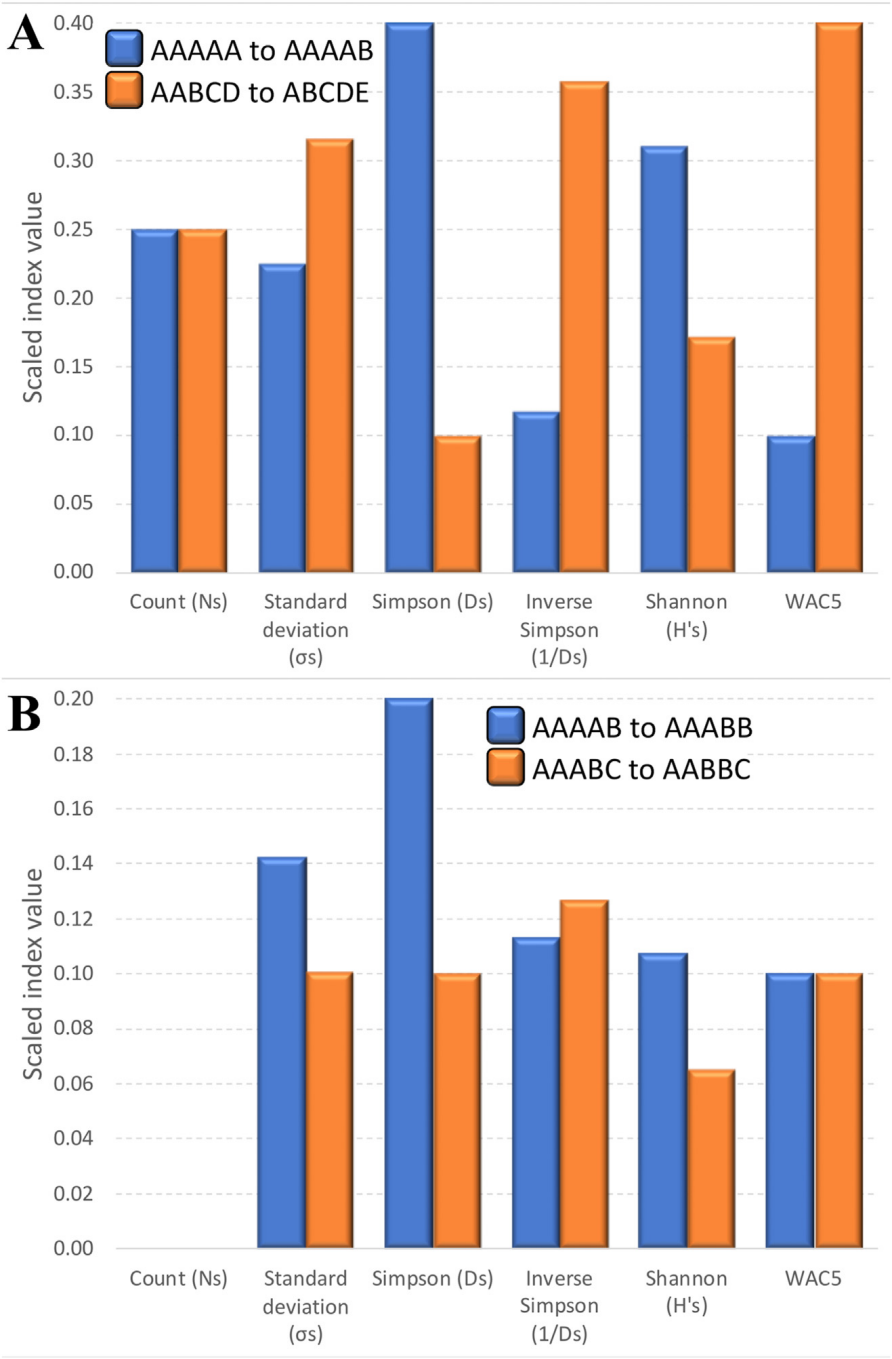
Absolute changes in the scaled values of six indices. A. The change when the competitive balance increases from the minimal value (0.0) to the next step (AAAAA to AAAAB) and when it increases from the penultimate step to the maximal value of 1.0 (AABCD to ABCDE). B. The change when the total number of champions remains the same, but the distribution of wins shifts. The AAAAB to AAABB step shows the change in values when the total number of champions is *N* = 2, while the AAABC to AABBC step shows the change in values when the total number of champions is *N* = 3.

Scaled values of all tested indices increased in the order AAAAA, AAAAB, AAABB, AAABC, AABBC, AABCD, ABCDE, with the exception of the index based on counts (*N*_*s*_), which showed identical values for AAAAB and AAABB (0.25), and for AAABC and AABBC (0.50) (Figure 1). The largest increase in the index values between two successive combinations of champions (when ordered from the smallest to the largest competitive balance) were observed for the AAAAA to AAAAB step when using *D*_*s*_ (0.40) and *H'*_*s*_ (0.31), and for the AABCD to ABCDE step when using WAC_5_ (0.40), *1/D*_*s*_ (0.36), and *σ*_*s*_ (0.32). Four pairwise differences had value of 0.25 when calculated using *N*_*s*_ (Figure 2a). These results imply that *D*_*s*_ and *H'*_*s*_ may be preferred for analyses when the minimum competitive balance (AAAAA) needs to be accentuated, while WAC_5_*, 1/D*_*s*_, and *σ*_*s*_ may be more preferable for the analyses that emphasize the maximum competitive balance (the lowest concentration of championships) (ABCDE). There was a substantial difference among indices in their sensitivity to the change in distribution of championship wins when the number of teams that won those championships (*N*) was constant, i.e., differences between AAAAB and AAABB (both *N* = 2), and between AAABC and AABBC (both *N* = 3) (Figure 2b). The change was larger between AAAAB and AAABB than between AAABC and AABBC when using *σ*_*s*_, *D*_*s*_, and *H'*_*s*_; smaller when applying 1/*D*_*s*_, and equal when using WAC_5_. No effect was recorded, of course, for *N*_*s*_ that considers only the number of different champions, not the distribution of their wins. In summary, when compared to other tested indices, WAC_5_ gives the most weight (0.40) to the final step that leads to the maximum competitive balance (from AABCD = 0.60 to ABCDE = 1.00) while having an equal sensitivity (0.10) to the change in distribution of wins among champions at *N* = 2 (AAAAB = 0.10, AAABB = 0.20) and *N* = 3 (AAABC = 0.30, AABBC = 0.40).

## 5 Data analysis

### 5.1 WAC_5_ and aWAC_5_

The WAC_5_ index indicates competitive balance (through concentration of championships) within a 5-year period. Individual WAC_5_ values calculated from subsequent sets of years (e.g., sliding window for years 1–5, then for years 2–6) are not, however, fully independent from each other because champions in years 2–5 would be used in both calculations. To preserve the relationship between subsequent WAC_5_ values when estimating confidence intervals for ‘average WAC_5_’ (aWAC_5_), the stationary block bootstrapping approach [32, 33] has been applied. This non-parametric, model-free approach divides data into blocks that are resampled with replacement and the new data series are then used to determine confidence intervals. The ‘tsboot’ and ‘boot.ci’ functions in R's ‘boot’ package [34, 35] were used to perform 100,000 resampling runs for each analyzed competition and to determine confidence intervals, respectively.

### 5.2 eWAC_5_ and ΔWAC_5_

Because each WAC_5_ is calculated from only five consecutive years (championships, tournaments), aWAC_5_ values cannot distinguish between the maximum competitive balance (aWAC_5_ = 1.0) reached through repeated wins of the same five champions (e.g., ABCDE-ABCDE over a 10-year evaluation period), or the one reached through wins of ten unique champions (ABCDE-FGHIJ). However, the actual number of champions and the frequency of their wins can be permutated over years and this new distribution of championship wins can be subsequently used to calculate WAC_5_ values. The average WAC_5_ values calculated from a large number of permutations are thus based on the random distribution of actual champions over the whole evaluation period and provide an effective statistical parameter that combines information about the number of champions and the frequency of their wins. This new index, termed ‘expected WAC_5_’ (eWAC_5_), can distinguish between the combinations of champions described in the above example (20,000 permutations yielded eWAC_5_ value of 0.604 for ABCDE-ABCDE, while ABCDE-FGHIJ has always value of 1.0). The difference between eWAC_5_ and aWAC_5_ values (ΔWAC_5_ = eWAC_5_ - aWAC_5_) in each competition indicates a relative grouping of the champions' wins in aWAC_5_ as compared to their random distribution over years (eWAC_5_). Higher, positive values of the index are suggestive of a larger relative grouping of teams' wins. It needs to be emphasized, however, that the grouping of championship wins identified through this parameter for a five-year period is relative (based on the comparison with random distribution of wins), not absolute (e.g., actual count of wins by a champion in a sequence). This value is expected to be larger for sports involving individual athletes, as active careers of athletes are typically shorter than existence of sport teams, thus championship wins of individual athletes are more likely to be grouped together. Alternatively, this parameter could be calculated from logit transformed eWAC_5_ and aWAC_5_ values thus stretching both tails of their distributions. eWAC_5_ values for each evaluated competition were calculated from 10,000 permutations performed with Microsoft Excel for Mac v. 16.16.21 (Microsoft, Redmond, WA, USA).

### 5.3 Change in competitive balance between eras

To evaluate changes in competitive balance, the 1960 to 2020 period has been split into two eras, 1960 to 1989, and 1990 to 2020. Only competitions with at least 44 championships during this period were considered for the analysis, therefore 13 out of 68 competitions were eliminated. On the other hand, seven new datasets that combine WAC_5_ values from multiple competitions were created. Such datasets can be used to identify the overall trends in competitive balance for certain sports, leagues, or geographic areas. These seven datasets were: N.Am-4 (combined results from four most economically important, professional leagues in North America: B-NBA, Ba-MLB, F-NFL, and IH-NHL), Euro-T5 (combined results from the top five European soccer leagues: S-Eng, S-Fra, S-Ger, S-Ita, and S-Spa), Euro-T5-C (combined cup competition results from the top five European soccer leagues: S-Eng-C, S-Fra-C, S-Ger-C, S-Ita-C, and S-Spa-C), Euro-Oth (combined results from all other European soccer leagues analyzed in this study), Euro-Oth-C (combined cup competition results from all other European soccer leagues analyzed in this study), T-Men (combined results of tennis Grand Slam tournaments for men: T-Aus, T-Eng, T-Fra, T-USA), and T-Women (combined results of tennis Grand Slam tournaments for women: T-Aus-W, T-Eng-W, T-Fra-W, T-USA-W). The significance of differences between WAC_5_ values for the two eras were determined using 100,000 bootstrapping. The monotonic trends in competitive balances were tested using the block bootstrapping version of the Mann–Kendall non-parametric test that is suitable for serially correlated (autocorrelated) data [36]. The ‘bbsmk’ function in R's ‘modifiedmk’ package [35, 37] was used to perform 50,000 resampling runs for each analyzed competition to determine the significance of Mann-Kendall *τ* statistics.

## 6 Results

### 6.1 Evaluation of competitive balance using aWAC_5_ values

**Figure 3:**
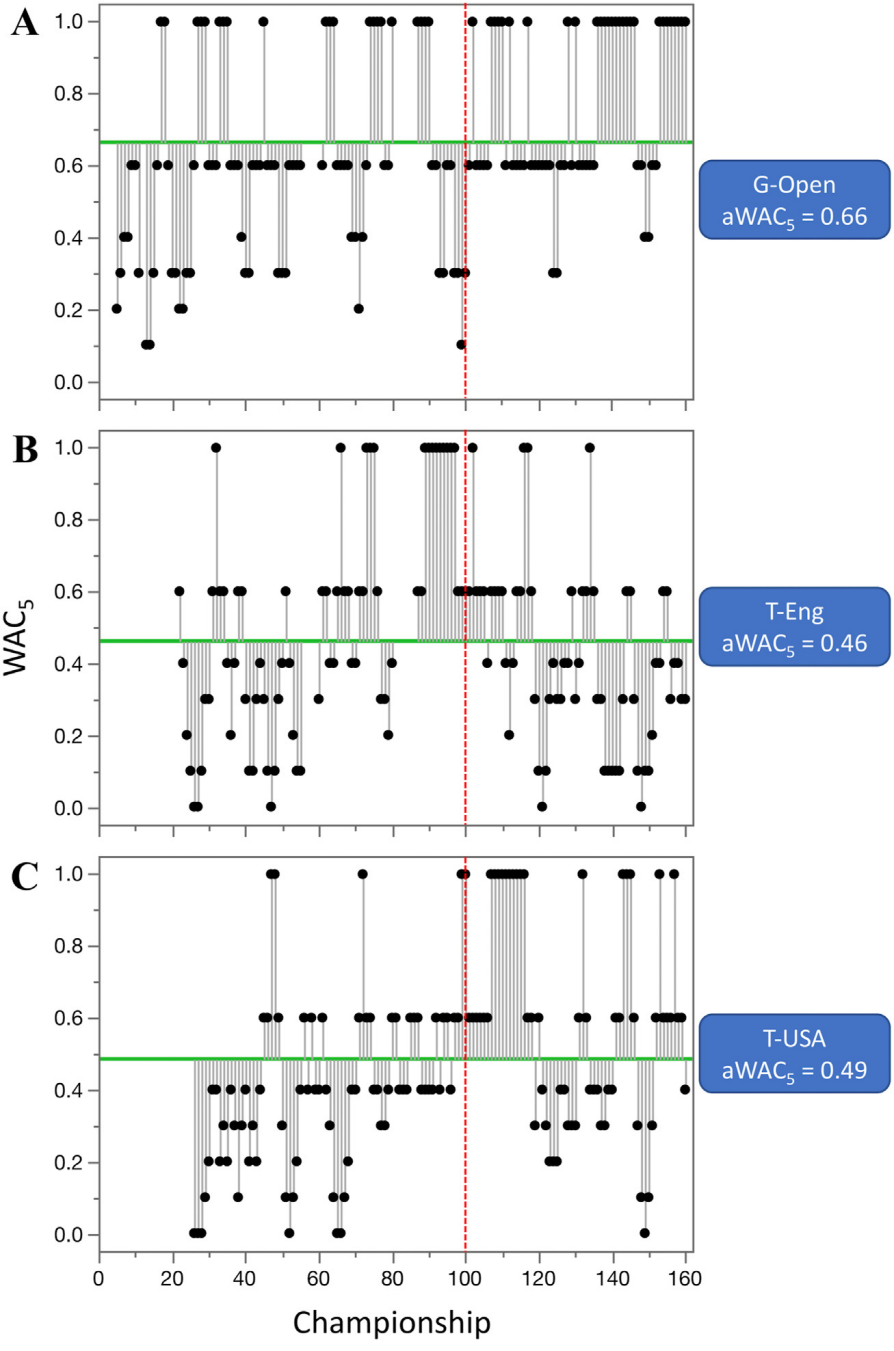
Historical outline of competitive balance for individual sports with the longest range of years. The oldest tournaments are: A. golf—British Open (G-Open), B. tennis—Wimbledon (T-Eng), and C. tennis—US Open (T-USA). Higher WAC_5_/aWAC_5_ values indicate a higher competitive balance. The black dots and the lines connected with them show WAC_5_ values for each championship (year) and their distance to aWAC_5_, respectively. The aWAC_5_ value for each competition is indicated by the green, horizontal line. The vertical red lines show year 1960, the beginning of the period that was used for detailed analysis of competitive balance.

**Figure 4:**
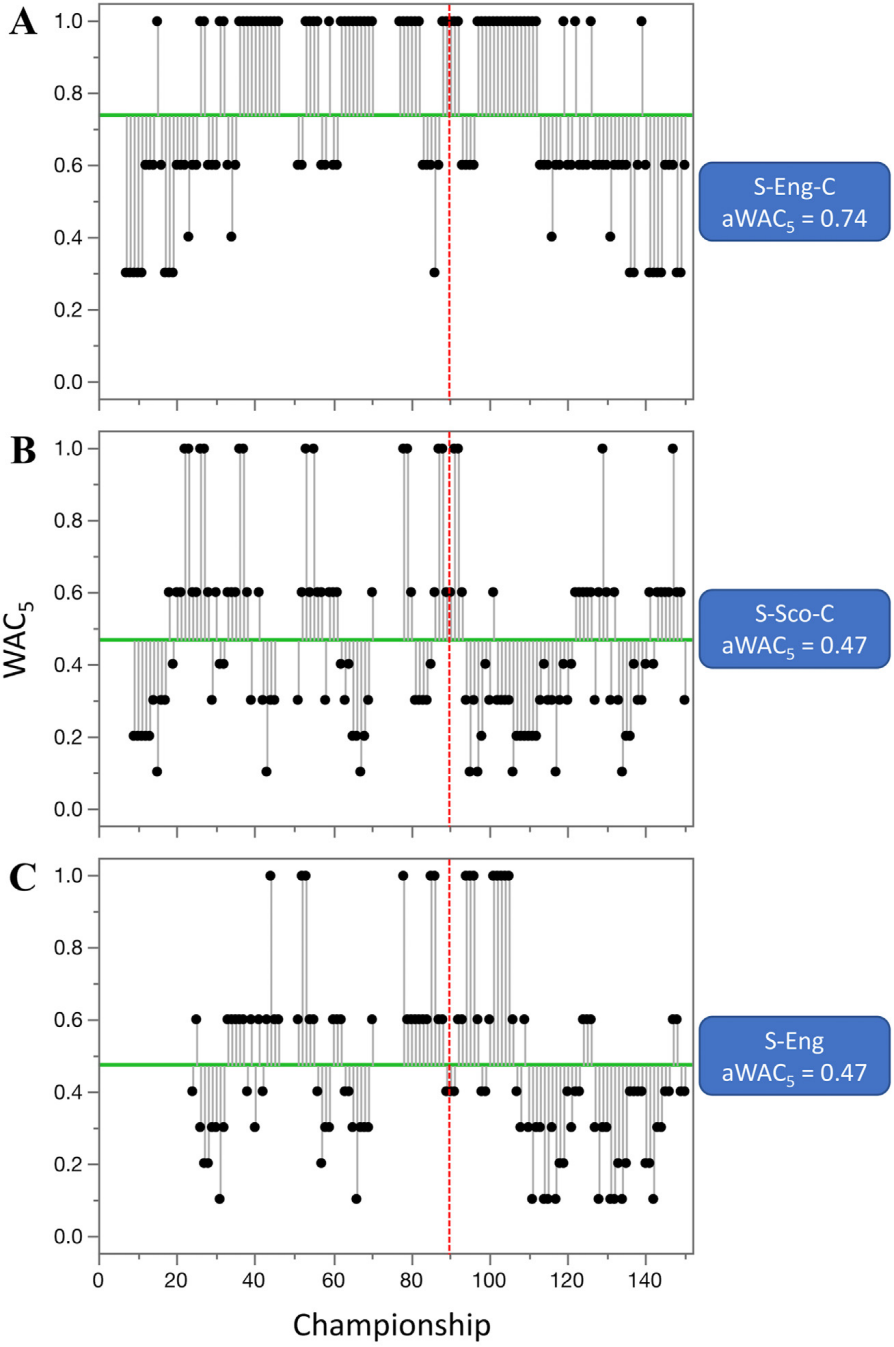
Historical outline of competitive balance for team sports with the longest range of years. The oldest competition are: A. soccer—English Football Association Cup (S-Eng-C), B. soccer—Scottish Cup (S-Sco-C), and C. soccer - English league (S-Eng). Higher WAC_5_/aWAC_5_ values indicate a higher competitive balance. The black dots and the lines connected with them show WAC_5_ values for each championship (year) and their distance to aWAC_5_, respectively. The aWAC_5_ value for each competition is indicated by the green, horizontal line. The vertical red lines show year 1960, the beginning of the period that was used for detailed analysis of competitive balance.

Competitive balance was analyzed on the set of 68 (mostly) annual competitions. The three oldest competitions included in analyses were golf—British Open (G-Open, years 1870–2019), tennis—Wimbledon (T-Eng, 1877–2019), and tennis—US Open (T-USA, 1881–2019) for individual sports, while for the team competitions they were soccer—English Football Association Cup (S-Eng-C, 1872–2019), soccer—Scottish Cup (S-Sco-C, 1874–2019), and soccer—English Premier League (S-Eng, 1889–2019) (Figures 3 and 4, Table 1). For the period starting at 1960, the average WAC_5_ (aWAC_5_) ranged from 0.13 for women ice hockey World Championship and Olympic Games (IH-WC-W) to 0.94 for soccer - UEFA Cup Winners' Cup (S-CWC) (Table 2).

**Table 2:** Parameters of competitive balance calculated for 68 competitions.

Competition	eWAC_5_	aWAC_5_			ΔWAC_5_	
	Mean	Mean	CI95L	CI95H	Value	Signif.
B-NBA	0.56	0.34	0.26	0.43	0.22	∗∗∗
B-NCAA	0.81	0.71	0.52	0.85	0.11	
B-NCAA-W	0.63	0.47	0.38	0.57	0.16	∗∗∗
B-Spa	0.27	0.23	0.13	0.34	0.04	
Ba-Jap	0.60	0.44	0.30	0.59	0.17	∗∗
Ba-Mex	0.59	0.56	0.48	0.64	0.03	
Ba-MLB	0.86	0.68	0.58	0.78	0.18	∗∗∗
C-WC	0.29	0.28	0.22	0.36	0.02	
C-WC-W	0.44	0.39	0.30	0.49	0.05	
F-AFL	0.71	0.51	0.45	0.57	0.20	∗∗∗
F-NFL	0.82	0.63	0.55	0.71	0.20	∗∗∗
F1	0.88	0.51	0.41	0.62	0.36	∗∗∗
F1-E	0.59	0.23	0.18	0.29	0.36	∗∗∗
G-Mas	0.92	0.75	0.66	0.84	0.16	∗∗∗
G-Open	0.94	0.77	0.68	0.86	0.17	∗∗∗
G-PGA	0.95	0.86	0.79	0.93	0.09	∗∗∗
G-US	0.96	0.90	0.84	0.96	0.06	∗∗
IH-NHL	0.71	0.44	0.29	0.62	0.26	∗∗∗
IH-Rus	0.43	0.27	0.14	0.41	0.17	∗∗
IH-Svk	0.49	0.35	0.30	0.40	0.14	∗∗∗
IH-Swe	0.67	0.45	0.33	0.55	0.22	∗∗∗
IH-WC	0.43	0.33	0.22	0.44	0.10	∗
IH-WC-W	0.16	0.13	0.09	0.18	0.03	
R-Eng	0.52	0.33	0.25	0.42	0.19	∗∗∗
R-Fra	0.71	0.50	0.38	0.63	0.21	∗∗∗
R–I&NI	0.58	0.47	0.36	0.58	0.12	∗
R-Ken	0.49	0.23	0.18	0.28	0.26	∗∗∗
S-Bel	0.41	0.34	0.29	0.39	0.06	∗∗
S-Bel-C	0.73	0.70	0.62	0.77	0.03	
S-Bra	0.75	0.56	0.45	0.68	0.19	∗∗∗
S-CL	0.75	0.60	0.47	0.72	0.15	∗∗
S-CL-C	0.53	0.43	0.30	0.56	0.10	
S-CSR	0.55	0.31	0.23	0.41	0.23	∗∗∗
S-CSR-C	0.58	0.62	0.53	0.73	-0.05	
S-CWC	0.95	0.94	0.86	1.00	0.00	
S-CWC-C	0.64	0.68	0.58	0.80	-0.04	
S-Cze	0.37	0.28	0.17	0.39	0.09	
S-Cze-C	0.59	0.54	0.43	0.66	0.04	
S-EL	0.90	0.76	0.65	0.85	0.14	∗∗∗
S-EL-C	0.60	0.45	0.35	0.56	0.14	∗∗
S-Eng	0.62	0.43	0.31	0.58	0.19	∗∗
S-Eng-C	0.73	0.70	0.58	0.83	0.03	
S-Fra	0.69	0.42	0.33	0.52	0.26	∗∗∗
S-Fra-C	0.78	0.66	0.60	0.73	0.12	∗∗∗
S-Ger	0.49	0.45	0.34	0.57	0.04	
S-Ger-C	0.67	0.63	0.52	0.74	0.04	
S-Ita	0.44	0.41	0.30	0.51	0.03	
S-Ita-C	0.70	0.64	0.53	0.75	0.06	
S-MLS	0.76	0.63	0.56	0.70	0.13	∗∗∗
S-NCAA	0.79	0.54	0.42	0.65	0.25	∗∗∗
S-Net	0.35	0.30	0.25	0.37	0.05	
S-Net-C	0.63	0.58	0.47	0.71	0.05	
S-Por	0.26	0.21	0.16	0.25	0.06	∗∗
S-Por-C	0.52	0.50	0.42	0.57	0.02	
S-Sco	0.26	0.19	0.13	0.26	0.07	∗∗
S-Sco-C	0.47	0.41	0.32	0.51	0.06	
S-Spa	0.34	0.29	0.22	0.35	0.05	∗
S-Spa-C	0.59	0.64	0.54	0.73	-0.05	
S-Svk	0.50	0.34	0.27	0.40	0.16	∗∗∗
S-Svk-C	0.61	0.61	0.50	0.72	-0.01	
T-Aus	0.86	0.47	0.37	0.57	0.39	∗∗∗
T-Aus-W	0.84	0.41	0.32	0.50	0.43	∗∗∗
T-Eng	0.84	0.41	0.32	0.51	0.42	∗∗∗
T-Eng-W	0.80	0.40	0.31	0.48	0.40	∗∗∗
T-Fra	0.84	0.49	0.37	0.61	0.34	∗∗∗
T-Fra-W	0.88	0.59	0.48	0.70	0.29	∗∗∗
T-USA	0.90	0.59	0.46	0.72	0.31	∗∗∗
T-USA-W	0.86	0.46	0.36	0.56	0.39	∗∗∗

eWAC_5_: expected values of WAC_5_; aWAC_5_: observed average values of WAC_5_; ΔWAC_5_: difference between the expected and observed values of WAC_5_ (ΔWAC_5_ = eWAC_5_ - aWAC_5_). Higher values of aWAC_5_ and eWAC_5_ indicate a higher competitive balance. Higher positive values of ΔWAC_5_ indicate a higher relative grouping of champions.

CI95L and CI95H: the lower and upped bounds of the 95% confidence intervals.

Asterisks indicate p-values for ΔWAC_5_: ∗p < 0.1 (suggestive), ∗∗p < 0.05 (significant), ∗∗∗p < 0.01 (highly significant).

The exceptionally low competitive balance of IH-WC-W was caused by a very limited number of teams that won championships, with only two national teams winning all of them, Canada 14 and USA 11. Competitive balance was higher in the men's ice hockey championships reaching aWAC_5_ of 0.33. Four ice hockey leagues had aWAC_5_ values from 0.27 (IH-Rus, Russia), through 0.35 (IH-Svk, Slovakia), to 0.44 (IH-NHL, USA/Canada) and 0.45 (IH-Swe, Sweden). Low aWAC_5_ at IH-Rus has been likely influenced by CSKA Moscow winning 45.0% of all championships (27 out of 60), with 25 out of them in the 30-year period (from 1960 to 1990). In basketball, the lowest aWAC_5_ values were detected for B-Spa (Spain, 0.23) where majority of championships (50 out of 60, 83.3%) were won by either Real Madrid (33) or FC Barcelona (17). Competitive balance was higher at B-NBA (USA/Canada, 0.34), B-NCAA-W (USA women colleges, 0.47), and particularly at B-NCAA (USA men colleges, 0.71). In the latter two, college championships, the champions were determined in the play-off tournaments with only a single-elimination game between two matched-up teams. This kind of a knockout tournament may lead to more frequent elimination of favorites. The WAC_5_ values for baseball ranged from 0.44 (Ba-Jap, Japan), through 0.56 (Ba-Mex, Mexico), to 0.68 (Ba-MLB, USA/Canada). The lower aWAC_5_ for Ba-Jap is likely caused by 18 championship wins of Yomiuri Giants, nine of them between 1965 and 1973. Low levels of competitive balance were determined for curling when considering either men's (C-WC, 0.28) or women's (C-WC-W, 0.39) teams. World curling championships were dominated by Canada in both categories with 58.3% of championships won (35 out of 60) in the men's category, and 41.4% of championships won (17 out of 41) in the women's category. American football (F-NFL, USA) and Australian rules football (F-AFL, Australia) leagues reached aWAC_5_ of 0.63 (F-NFL) and 0.51 (F-AFL), respectively. Reigning champions defended their titles in only 15.1% of championships in F-NFL (8 out of 53), and in 16.7% championships in F-AFL (10 out of 60). Rugby championships have had aWAC_5_ values in the range from 0.23 (R-Ken, Kenya) to 0.50 (R-Fra, France). Low values at R-Ken were likely caused by frequent wins of reigning champions that happened in 53.1% of championships (26 out of 49).

**Figure 5:**
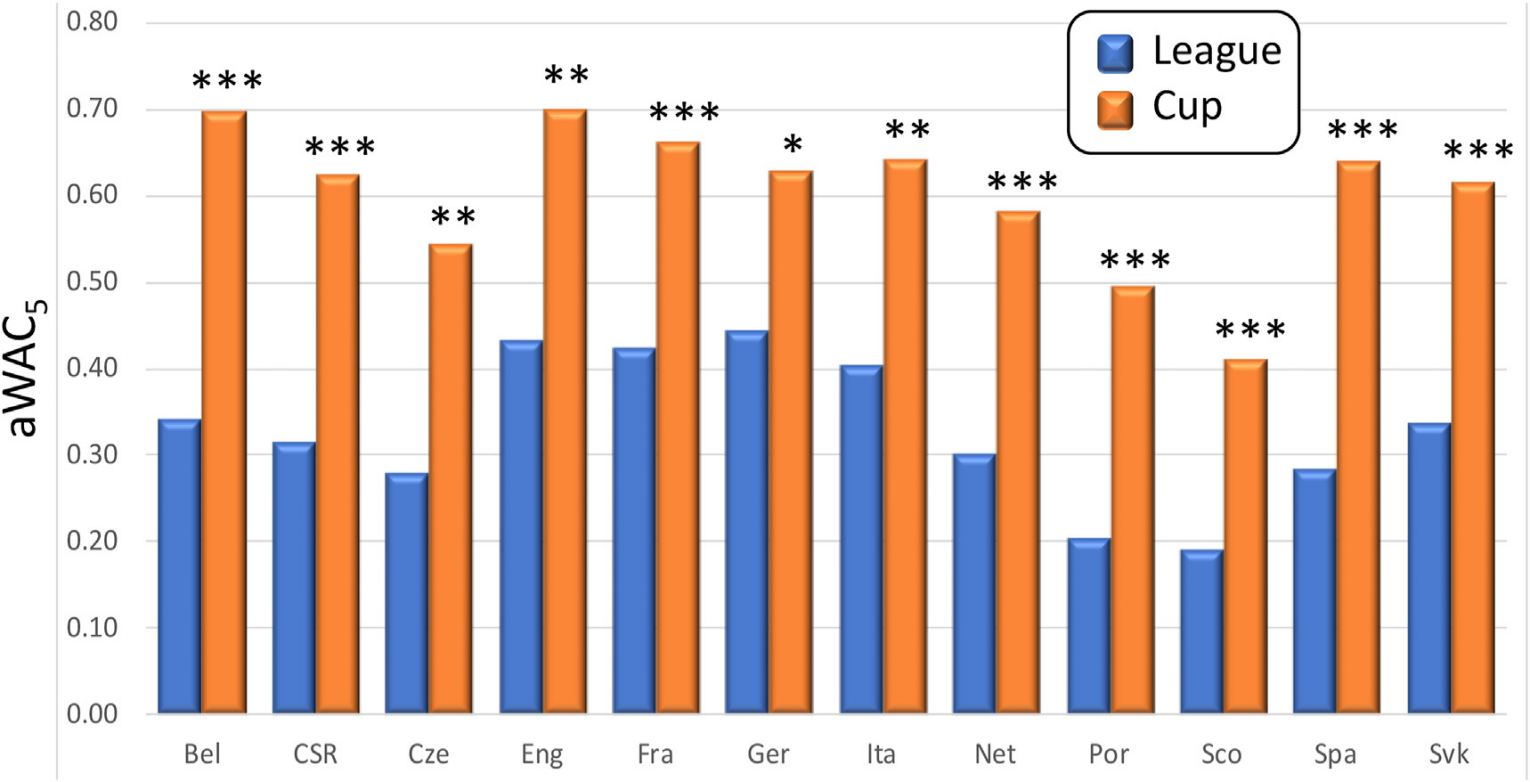
Comparison of competitive balance across 12 European soccer leagues and domestic cup competitions. Higher aWAC_5_ values indicate a higher competitive balance. Abbreviations for counties/federations are as follows: Bel—Belgium, CSR—former Czechoslovakia, Cze—Czechia, Eng—England, Fra—France, Ger—Germany, Ita—Italy, Net—The Netherlands, Por—Portugal, Sco—Scotland, Spa—Spain, and Svk—Slovakia. Asterisks indicate the significance of difference between the pairs of competitions within each country/federation: ∗p < 0.1 (suggestive), ∗∗p < 0.05 (significant), ∗∗∗p < 0.01 (highly significant).

Among soccer competitions, the highest aWAC_5_ value (0.94) was detected for UEFA Cup Winners' Cup (S-CWC, Table 2). A high aWAC_5_ value for this (already defunct) international competition was expected, because only teams winning their domestic cup competition could participate in it. In addition, when S-CWC champions qualified for a more prestigious UEFA Champions League (S-CL) competition, they have not been defending their S-CWC title. Comparison among top three (former and current) European cups competitions showed aWAC_5_ increasing from 0.60 for S-CL, through 0.76 for UEFA Europa League (S-EL), to 0.94 for S-CWC. When federations (or countries) of the champions were considered, the WAC_5_ values were justifiably lower, because multiple champions could originate from the same federation. However, the aWAC_5_ values for federations (countries) increased in the same order as for individual teams, from 0.43 for S-CL-C, through 0.45 for S-EL-C, to 0.68 for S-CWC-C. The lowest aWAC_5_ value among European leagues was detected at Scotland (S-Sco, 0.19), while the highest one was found at Germany (S-Ger, 0.45). The low competitive balance in S-Sco was due to a high frequency of championships won by only two teams, Celtic FC (50.0%, 30 out of 60) and Rangers FC (38.3%, 23 out of 60). The aWAC_5_ values for non-European, soccer championships were higher than the highest one found at European leagues (S-Ger, 0.45) and reached 0.54 at S-NCAA (USA, amateur, college competition with single-elimination tournament), 0.56 at S-Bra (Brazil), and 0.63 at S-MLS (USA/Canada where champions were determined in play-off elimination). When domestic cup competitions were compared across European federations (countries), the lowest competitive balance was detected again at Scotland (S-Sco-C = 0.41) and the highest one at England (S-Eng-C = 0.70). The comparison of the two soccer competitions (league and cup) within each country showed a substantial difference between their aWAC_5_ values (Figure 5). In every federation the values for leagues were lower with the difference ranging from 0.19 in Germany (0.45 S-Ger and 0.63 S-Ger-C, p < 0.1) to 0.36 in Spain (0.29 S-Spa and 0.64 S-Spa-C, p < 0.01). Because in every federation, teams from the top league are allowed to compete, and can potentially win both domestic competitions (frequently called ‘double’), the very large difference detected between aWAC_5_ values could seem unexpected. This difference may be caused by several factors, including the competition format and the team's preference or aspiration. While all analyzed leagues were played in a round-robin format (with occasional modifications), cup competitions were played as a knockout tournament with direct-elimination. Thus, loss led to immediate elimination (with exceptions when more than a single match was played) in cup competitions but not in leagues. Moreover, teams from lower tiers of domestic leagues see cup competitions as an opportunity to prove their worth against top teams (so called ‘giant slayers’). Furthermore, top teams may have had a very busy schedule when also playing in a European cup competition, and therefore they could have rested a few or more of their best players.

In competitions involving individual athletes, the aWAC_5_ values ranged from 0.40 (women tennis at Wimbledon, T-Eng-W) to 0.90 (golf at US Open, G-US). All four golf tournaments have had very high aWAC_5_ values ranging from 0.75 for G-Mas (Masters Tournament) to 0.90 for G-US, placing them into top six of the 68 evaluated competitions (Table 2). In the 1960–2020 period, the reigning champions were able to defend their titles only at the rate of 3.3% (2 out of 60) at G-US, 5.0% (3 out of 60) at G-Mas and G-PGA (PGA Championship), and 8.3% (5 out of 60) at G-Open (British Open). For the four tennis, Grand Slam tournaments the aWAC_5_ values in men's category ranged from 0.41 (Wimbledon, T-Eng) to 0.59 (US Open, T-USA) and in women's category from 0.40 (T-Eng-W) to 0.59 (French Open, T-Fra-W). There was no obvious difference in the aWAC_5_ values between the two categories, and in both of them the lowest competitive balance was observed at Wimbledon (T-Eng-W, 0.40; T-Eng, 0.41), followed by Australian Open (T-Aus-W, 0.41; T-Aus, 0.47). The aWAC_5_ value of 0.51 was detected for Formula One World Drivers' Championship (F1) but only 0.23 for engines used in F1 racing cars (F1-E). This difference in values reflects that 60 championships were won by 29 drivers, but only 10 different engines were used in the winning race cars.

### 6.2 Change in competitive balance from 1960—1989 to 1990–2020

The comparison of aWAC_5_ values calculated for two eras, the earlier one (1960–1989) and the later one (1990–2020), was performed at 55 competitions that have had data for at least 44 championships or tournaments plus seven datasets that combine WAC_5_ from multiple competitions (Table 3).

**Table 3:** Change in competitive balance from 1960—1989 to 1990–2020 as determined by differences in aWAC_5_ values.

Competition	aWAC_5_				Mann-Kendall	
	1960–1989	1990–2020	Difference	Signif. of Diff.	*τ*	Signif. of *τ*
B-NBA	0.31	0.37	0.06		0.08	
B-NCAA	0.62	0.79	0.18		0.17	
B-Spa	0.10	0.35	0.25	∗∗∗	0.50	∗∗∗
Ba-Jap	0.33	0.55	0.22		0.26	
Ba-Mex	0.59	0.53	-0.06		-0.03	
Ba-MLB	0.66	0.69	0.03		0.15	
C-WC	0.29	0.27	-0.02		0.11	
F-AFL	0.49	0.53	0.04		-0.01	
F-NFL	0.64	0.62	-0.03		0.08	
F1	0.65	0.38	-0.27	∗∗∗	-0.36	∗∗∗
F1-E	0.26	0.20	-0.07		-0.18	
G-Mas	0.74	0.77	0.03		0.14	
G-Open	0.70	0.84	0.14		0.17	
G-PGA	0.92	0.80	-0.12		-0.19	∗
G-US	0.96	0.85	-0.11	∗	-0.12	
IH-NHL	0.23	0.67	0.44	∗∗∗	0.49	∗∗
IH-Rus	0.10	0.43	0.33	∗∗∗	0.46	∗∗
IH-Swe	0.37	0.52	0.15		0.40	∗∗∗
IH-WC	0.20	0.45	0.25	∗∗∗	0.40	∗∗∗
R-Fra	0.45	0.56	0.11		0.09	
R-Ken	0.15	0.28	0.13	∗∗	0.33	∗∗∗
S-Bel	0.33	0.35	0.02		0.06	
S-Bel-C	0.69	0.71	0.02		0.05	
S-Bra	0.51	0.60	0.09		0.08	
S-CL	0.53	0.67	0.15		0.17	
S-CL-C	0.34	0.52	0.19		0.13	
S-Cze-C	0.44	0.63	0.19		0.26	∗∗
S-EL	0.80	0.74	-0.06		-0.22	∗
S-EL-C	0.58	0.39	-0.19	∗	-0.22	
S-Eng	0.53	0.34	-0.19	∗	-0.25	
S-Eng-C	0.85	0.55	-0.29	∗∗∗	-0.43	∗∗∗
S-Fra	0.42	0.43	0.01		-0.04	
S-Fra-C	0.68	0.64	-0.04		-0.12	
S-Ger	0.56	0.33	-0.24	∗	-0.43	∗∗∗
S-Ger-C	0.66	0.60	-0.06		-0.25	∗
S-Ita	0.49	0.31	-0.18	∗	-0.27	∗∗
S-Ita-C	0.68	0.61	-0.07		-0.26	∗∗
S-NCAA	0.44	0.62	0.18		0.32	∗∗
S-Net	0.34	0.26	-0.08		-0.19	
S-Net-C	0.61	0.55	-0.06		0.01	
S-Por	0.22	0.19	-0.04		-0.10	
S-Por-C	0.43	0.56	0.13		0.02	
S-Sco	0.26	0.12	-0.15	∗	-0.19	
S-Sco-C	0.33	0.50	0.17	∗	0.24	∗
S-Spa	0.27	0.30	0.02		0.12	
S-Spa-C	0.66	0.62	-0.04		-0.01	
S-Svk-C	0.54	0.67	0.13		0.17	
T-Aus	0.49	0.45	-0.03		-0.16	
T-Aus-W	0.38	0.45	0.07		0.31	∗∗
T-Eng	0.48	0.34	-0.14		-0.26	∗∗
T-Eng-W	0.40	0.40	0.00		-0.04	
T-Fra	0.54	0.44	-0.10		-0.32	∗∗
T-Fra-W	0.60	0.58	-0.02		-0.10	
T-USA	0.62	0.55	-0.08		-0.15	
T-USA-W	0.35	0.58	0.23	∗∗∗	0.22	∗
N.Am.-4^a^	0.43	0.59	0.15	∗∗∗	0.56	∗∗∗
Euro-T5^a^	0.46	0.34	-0.11	∗∗	-0.49	∗∗∗
Euro-T5-C^a^	0.70	0.61	-0.10	∗	-0.43	∗∗∗
Euro-Oth^a^	0.30	0.25	-0.05		-0.07	
Euro-Oth-C^a^	0.53	0.60	0.07		0.21	
T-Men^a^	0.53	0.44	-0.09		-0.35	∗
T-Women^a^	0.43	0.51	0.08		0.16	

Higher values of aWAC_5_ indicate a higher competitive balance. Positive difference shows a growing competitive balance in 1990–2020 as compared to the previous (1960–1989) period, while negative difference shows a decline in the competitive balance in the latter period (rounded values may not match exactly with the difference between two eras). Similarly, positive values of Mann-Kendall *τ* show an overall increasing trend in competitive balance, while negative values indicate a decreasing trend. Asterisks indicate p-values for differences and *τ*, respectively: ∗p < 0.1 (suggestive), ∗∗p < 0.05 (significant), ∗∗∗p < 0.01 (highly significant).

a Datasets that combine results from multiple competitions. Detailed information is provided in the section ‘Change in competitive balance between eras.’

**Figure 6:**
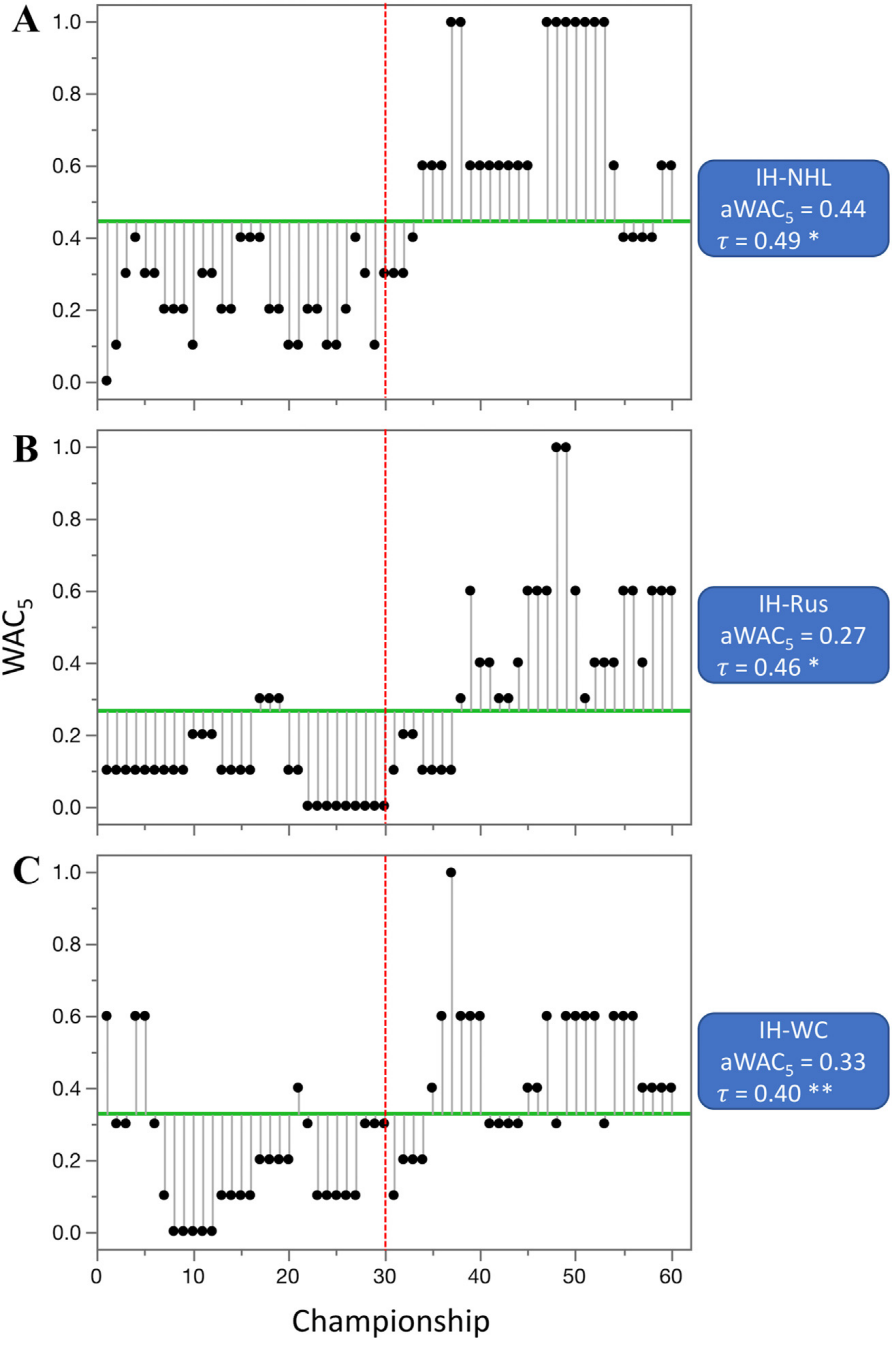
Competitions with the largest significant increase in competitive balance from 1960—1989 to 1990–2020. A. ice hockey—NHL (IH-NHL), B. ice hockey—Russia (IH-Rus), and C. ice hockey—World championships (IH-WC). Higher WAC_5_/aWAC_5_ values indicate a higher competitive balance. The black dots and the lines connected with them show WAC_5_ values for each championship (year) and their distance to aWAC_5_, respectively. The aWAC_5_ value for each competition is indicated by the green, horizontal line. The championship number 1 correspondents to year 1960, while the championship number 61 correspondents to year 2020. The vertical red lines separate the two evaluated eras. Asterisks indicate the significance of Mann-Kendall trend test (*τ*): ∗p < 0.1 (suggestive), ∗∗p < 0.05 (significant), ∗∗∗p < 0.01 (highly significant).

**Figure 7:**
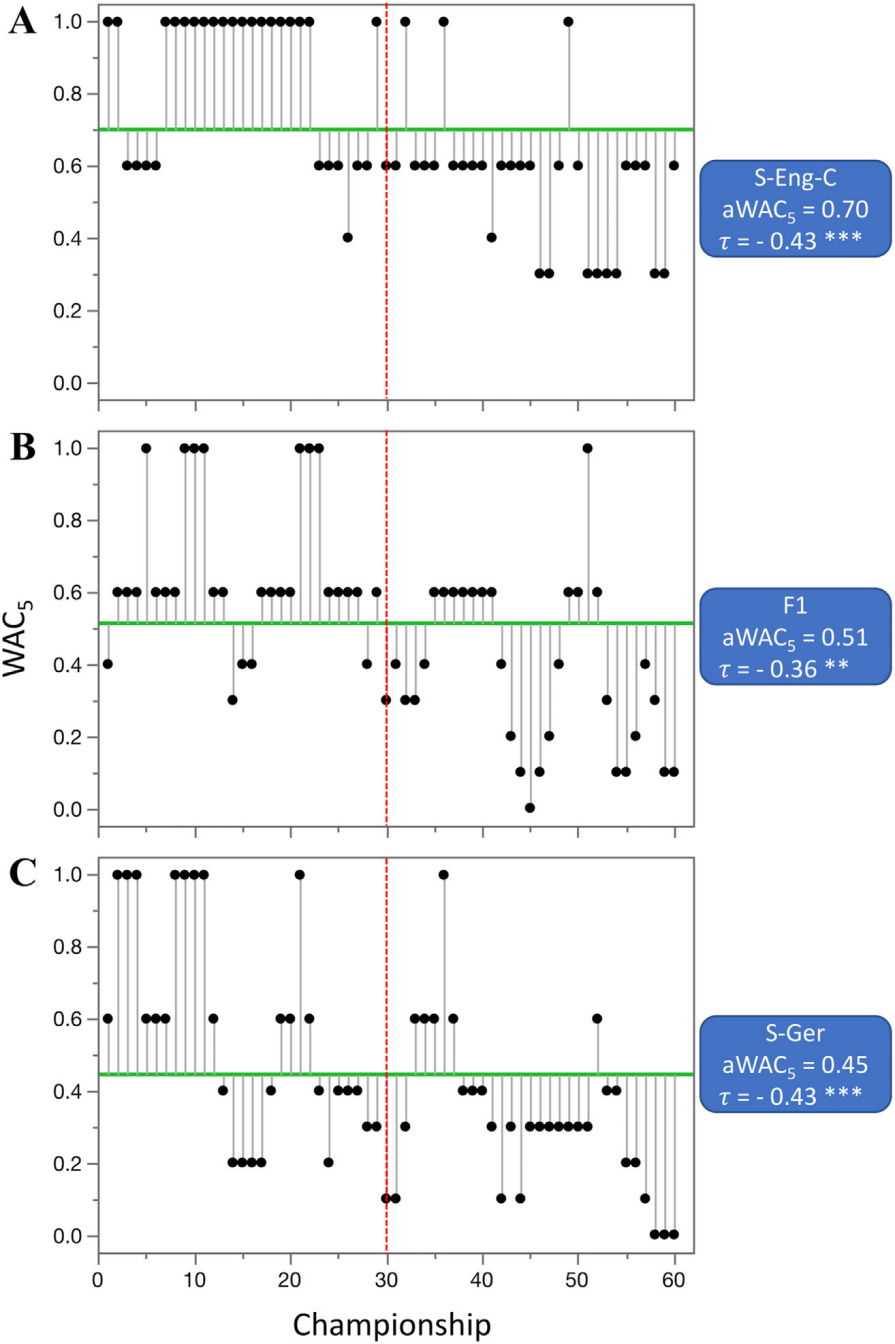
Competitions with the largest significant decrease in competitive balance from 1960—1989 to 1990–2020. A. soccer - English Football Association Cup (S-Eng-C), B. Formula One World Drivers' Championship (F1), and C. soccer—German league (S-Ger). Higher WAC_5_/aWAC_5_ values indicate a higher competitive balance. The black dots and the lines connected with them show WAC_5_ values for each championship (year) and their distance to aWAC_5_, respectively. The aWAC_5_ value for each competition is indicated by the green, horizontal line. The championship number 1 correspondents to year 1960, while the championship number 61 correspondents to year 2020. The vertical red lines separate the two evaluated eras. Asterisks indicate significance of the Mann-Kendall trend test (*τ*): ∗p < 0.1 (suggestive), ∗∗p < 0.05 (significant), ∗∗∗p < 0.01 (highly significant).

Out of 55 competitions that were evaluated (not including combined datasets), the aWAC_5_ values increased in 28 competitions, stayed at the same level in one, and decreased in 26. From the 28 competitions where aWAC_5_ increased, the difference was highly significant (p < 0.01) in five, significant (p < 0.05) in one, and suggestive (p < 0.1) in one of them. The largest increase in competitive balance, as indicated by the change in aWAC_5_ values, was observed for three ice hockey competitions; IH-NHL (increase of 0.44 from 0.23 to 0.67), IH-Rus (increase of 0.33 from 0.10 to 0.43), and IH-WC (increase of 0.25 from 0.20 to 0.45) (Figure 6), followed by the basketball league in Spain (B-Spa, increase of 0.25 from 0.10 to 0.35). The largest increase in individual sport competitions was detected at women's US Open tennis tournament (T-USA-W), from 0.35 to 0.58. From the 26 competitions where aWAC_5_ decreased from the earlier era, the difference was highly significant in two, and suggestive in six of them. The largest decrease in competitive balance was recorded for two soccer competitions and F1 racing; S-Eng-C (−0.29, from 0.85 to 0.55), F1 (−0.27, from 0.65 to 0.38), and S-Ger (−0.24, from 0.56 to 0.33) (Figure 7).

**Figure 8:**
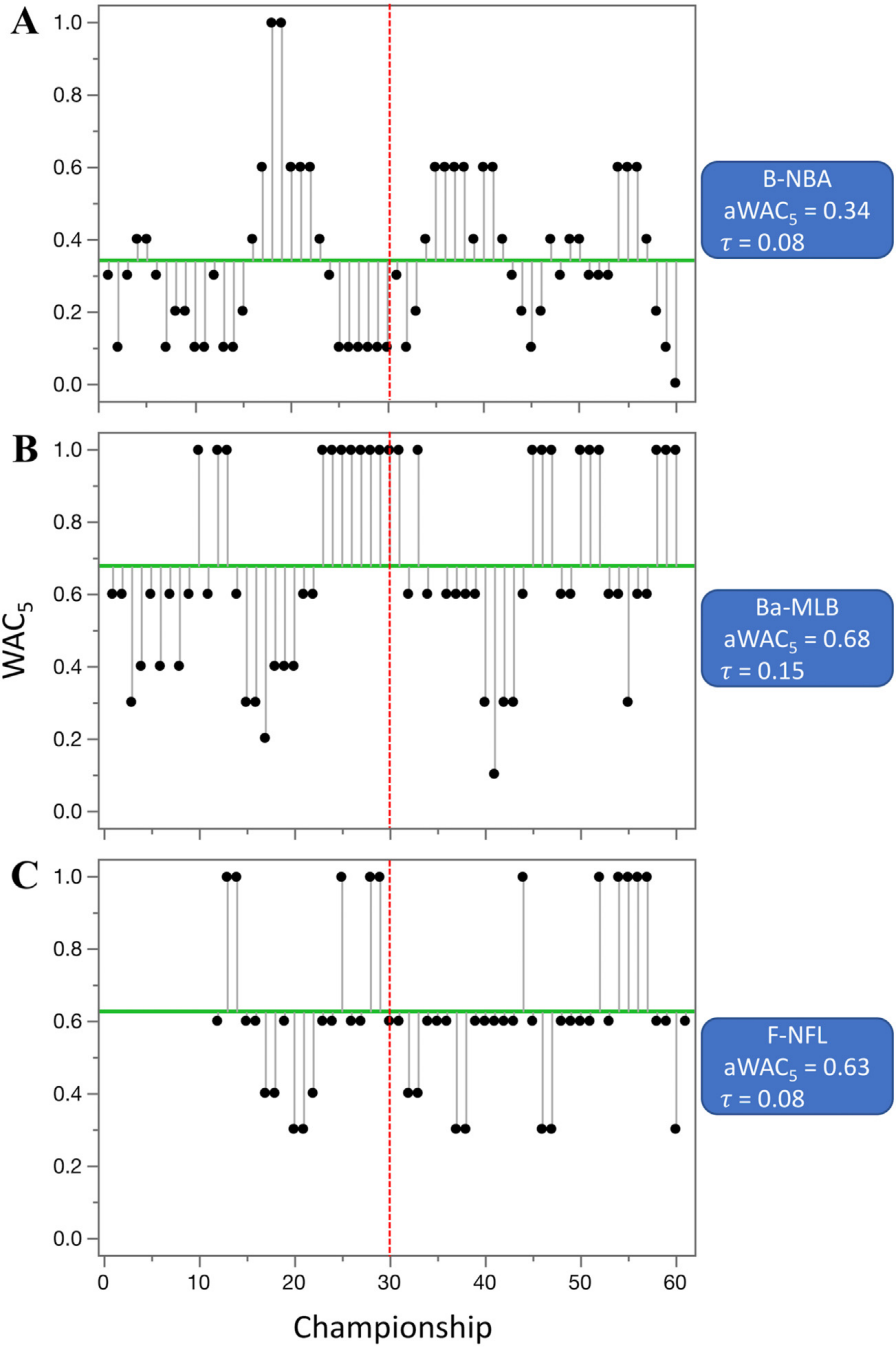
Three of the most popular North American, professional competitions with minimal change in competitive balance from 1960—1989 to 1990–2020. A. basketball (B-NBA), B. baseball (Ba-MLB), and C. football (F-NFL). Higher WAC_5_/aWAC_5_ values indicate a higher competitive balance. The black dots and the lines connected with them show WAC_5_ values for each championship (year) and their distance to aWAC_5_, respectively. The aWAC_5_ value for each competition is indicated by the green, horizontal line. The championship number 1 correspondents to year 1960, while the championship number 61 correspondents to year 2020. The vertical red lines separate the two evaluated eras. Asterisks indicate significance of the Mann-Kendall trend test (*τ*): ∗p < 0.1 (suggestive), ∗∗p < 0.05 (significant), ∗∗∗p < 0.01 (highly significant). Note that none of the tests was significant at p < 0.1.

**Figure 9:**
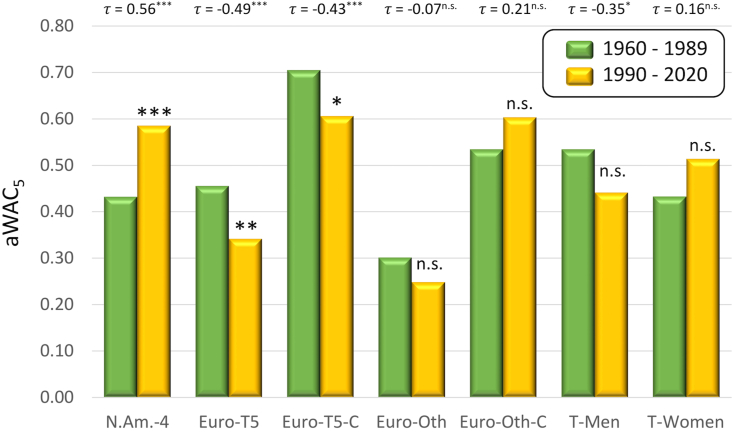
Change in competitive balance of seven datasets compiled from multiple competitions. N.Am-4 (four major, professional leagues in North America), Euro-T5 (top five European soccer leagues), Euro-T5-C (domestic cup competitions from top five European soccer leagues), Euro-Oth (all other European soccer leagues analyzed in this study), Euro-Oth-C (domestic cup competition results from all other European soccer leagues analyzed in this study), T-Men (combined results of tennis Grand Slam tournaments for men), and T-Women (combined results of tennis Grand Slam tournaments for women). Higher aWAC_5_ values indicate a higher competitive balance. The *τ* values of the Mann-Kendall trend test are shown above the graph. Asterisks indicate the significance of difference between two eras or the trend: ∗p < 0.1 (suggestive), ∗∗p < 0.05 (significant), ∗∗∗p < 0.01 (highly significant). Asterisks above columns show the significance for differences in aWAC_5_ values, those next to *τ* values for the Mann-Kendall trend test.

**Figure 10:**
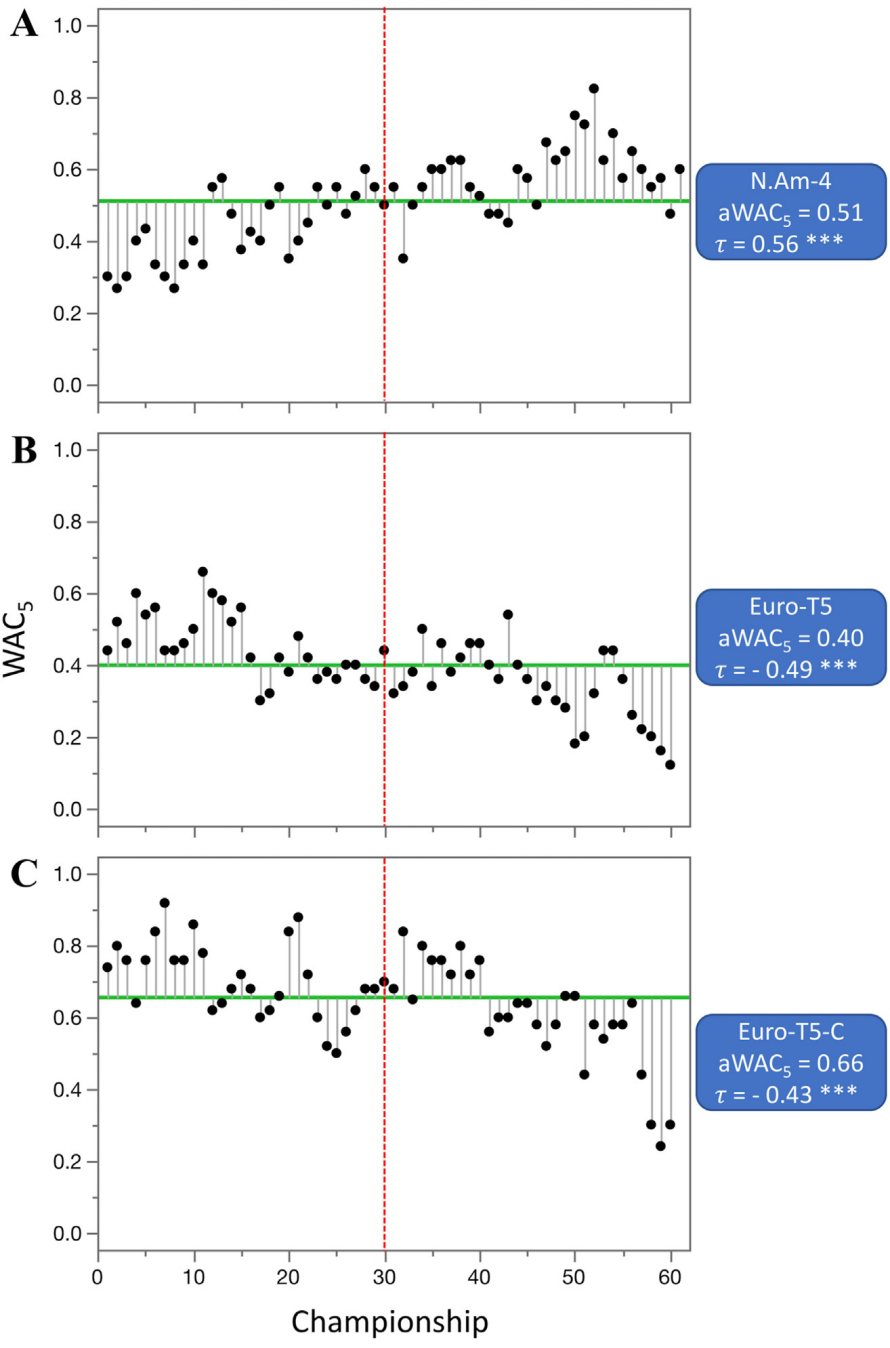
Three combined datasets with the largest change in competitive balance from 1960—1989 to 1990–2020. A. N.Am-4 (four major, professional leagues in North America), B. Euro-T5 (top five European soccer leagues), and C. Euro-T5-C (domestic cup competitions from top five European soccer leagues). Higher WAC_5_/aWAC_5_ values indicate a higher competitive balance. The black dots and the lines connected with them show WAC_5_ values for each championship (year) and their distance to aWAC_5_, respectively. The aWAC_5_ value for each competition is indicated by the green, horizontal line. The championship number 1 correspondents to year 1960, while the championship number 61 correspondents to year 2020. The vertical red lines separate the two evaluated eras. Asterisks indicate significance of the Mann-Kendall trend test (*τ*): ∗p < 0.1 (suggestive), ∗∗p < 0.05 (significant), ∗∗∗p < 0.01 (highly significant).

**Figure 11:**
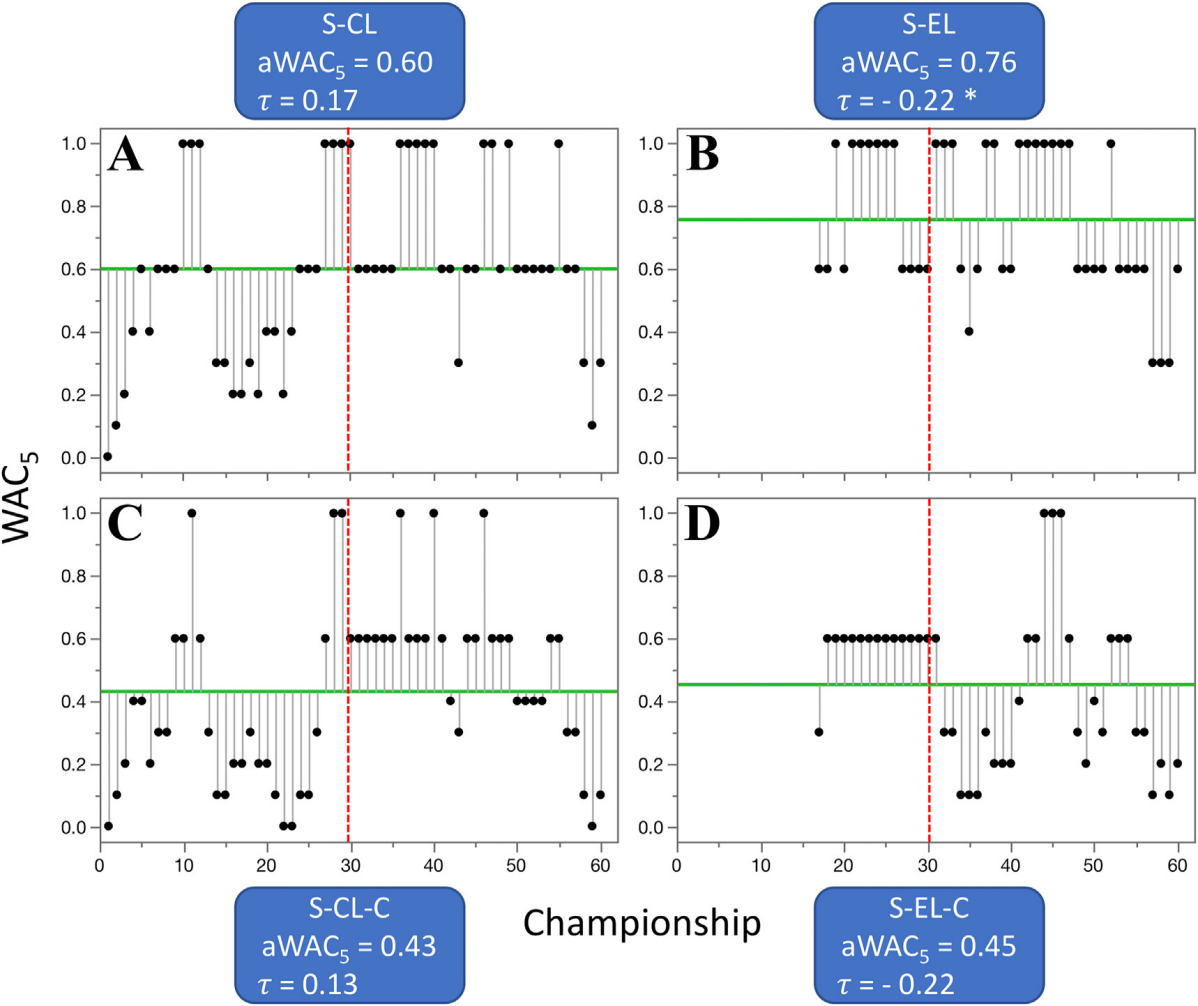
Changes in competitive balance of two European soccer's premier club competitions. A. and C. the Championship League (S-CL), and B. and D. the European League (S-EL). The top row (A. and B.) shows competitive balance calculated at the individual club level, while the bottom row (C. and D.) shows the results calculated at the country/federation level (when not the club itself, but country/federation of the champion was considered). Higher WAC_5_/aWAC_5_ values indicate a higher competitive balance. The black dots and the lines connected with them show WAC_5_ values for each championship (year) and their distance to aWAC_5_, respectively. The aWAC_5_ value for each competition is indicated by the green, horizontal line. The championship number 1 correspondents to year 1960, while the championship number 61 correspondents to year 2020. The vertical red lines separate the two evaluated eras, 1960–1989 from 1990 - 2020. Asterisks indicate significance of the Mann-Kendall trend test (*τ*): ∗p < 0.1 (suggestive), ∗∗p < 0.05 (significant), ∗∗∗p < 0.01 (highly significant).

Competitive balance changed only negligibly in three out of the five professional, North American competitions, with a large economic impact; increased by 0.06 in basketball (B-NBA, from 0.31 to 0.37), by 0.03 in baseball (Ba-MLB, from 0.66 to 0.69), and decreased by 0.03 in football (F-NFL, from 0.64 to 0.62) (Table 3, Figure 8). The increase in competitive balance (0.44) was highly significant in ice hockey (IH-NHL, from 0.23 to 0.67) (Table 3, Figure 6). The trend in competitive balance of soccer (S-MLS) has not been analyzed, because no data were available for the earlier era. When WAC_5_ values from the four leagues with data for both eras were combined together (N.Am-4), they indicated a highly significant, overall increase in competitive balance (from 0.43 to 0.59) (Table 3, Figures 9 and 10). The situation was substantially different in the top five European soccer leagues (Table 3), where the aWAC_5_ values decreased substantially in Germany (S-Ger, -0.24, from 0.56 to 0.33, p < 0.1), England (S-Eng, -0.19, from 0.53 to 0.34, p < 0.1), and Italy (S-Ita, -0.18, from 0.49 to 0.31, p < 0.1). A negligible increase in competitive balance was observed in Spain (S-Spa, 0.02, from 0.27 to 0.30) and France (S-Fra, 0.01, from 0.42 to 0.43). The combined values of aWAC_5_ from these five federations (Euro-T5) showed a substantial decline in competitive balance (−0.11, from 0.46 to 0.34, p < 0.05) (Figure 9). Similarly, when cup competitions at these five federations were pooled together (Euro-T5-C), a decline in competitive balance was observed (−0.10, from 0.70 to 0.61, p < 0.1) (Figure 9). In both combined datasets from the top five European soccer leagues and cup competitions the decline appeared to be more pronounced in recent years. The lowest aWAC_5_ values for the league competitions during last 60 years was observed in 2019 (0.12) and for the cup competitions in 2018 (0.24) (Figure 10). In comparison, combined aWAC_5_ values from other analyzed countries or federations (Euro-Oth, Euro-Oth-C) did not change significantly between the two eras (Table 3, Figure 9). Though the overall, 60-year mean of aWAC_5_ values from the top five federations was larger than the mean from other analyzed federations (Euro-T5 of 0.40 vs. Euro-Oth of 0.27, and Euro-T5-C of 0.66 vs. Euro-Oth-C of 0.36), the data from 2019 only showed a very different pattern (Euro-T5 of 0.12 vs. Euro-Oth of 0.30, and Euro-T5-C of 0.30 vs. Euro-Oth-C of 0.52). It is not possible to determine, however, if this is a long-term trend or only a temporary anomaly. When the change in competitive balance was evaluated at the European cups level, non-significant increase of 0.15 was observed for the Champions League (S-CL, from 0.53 to 0.67) and non-significant decrease of -0.06 for the Europa League (S-EL, from 0.80 to 0.74) (Table 3). A similar, but more pronounced pattern has been revealed then the competitive balance was calculated using federation of the champions. In such analysis, the competitive balance for the Champions League (S-CL-C) increased by 0.19 (from 0.34 to 0.52), while decreased by 0.19 (from 0.58 to 0.39, p < 0.1) for the Europa League (S-EL-C). Nevertheless, the last three years of the Championship League (2017–2019) suggest a declining trend in competitive balance compared to the earlier years of this era (1990–2020) (Figure 11). Pooled data for tennis Grand Slam tournaments did not show any significant changes in aWAC_5_ values, though there was an indication that the overall competitive balance in men's competitions (T-Men) might have decreased (from 0.53 to 0.44) while increasing (from 0.43 to 0.51) in women's competitions (T-Women) (Table 3, Figure 8).

The values of Mann-Kendall *τ* ranged from -0.43 (p < 0.01) for S-Ger to 0.50 (p < 0.01) for B-Spa (Table 3). A significant or suggestive decrease in competitive balance (negative values) was observed for 10 competitions, while an increase (positive values) was recorded for 11 competitions. Out of 25 competitions that were determined to have significant or suggestive change in competitive balance by at least one of the two employed methods (difference in aWAC_5_ and/or *τ*), four were identified only through differences in aWAC_5_, 10 were identified only when using the Mann-Kendall trend test, and 11 were detected by both methods. Generally, there was a strong linear correlation (r = 0.89, p < 0.0001) between the difference in aWAC_5_ values from two eras and the Mann-Kendall *τ* values.

### 6.3 Expected concentration (eWAC_5_) and relative grouping of champions (ΔWAC_5_)

The expected eWAC_5_ values were calculated from 10,000 permutations of the actual number and the frequency of champions reported for each of the 68 competitions. There was a positive, linear, significant correlation (r = 0.72, p < 0.001), between aWAC_5_ and eWAC_5_ values. The eWAC_5_ values were in the range from 0.16 for IH-WC-W to 0.96 for G-US (Table 2), indicating a very low and a very high balance in these two competitions. Similarly, as for aWAC_5_, very high eWAC_5_ values were determined for all golf competitions (0.92–0.96) and S-CWC (0.95). Contrasting, a very low balance of competition was found for several soccer leagues (S-Por, Portugal; S-Sco, Scotland; S-Spa, Spain; S-Net, The Netherlands; S-Cze, Czechia; and S-Bel, Belgium; from 0.26 to 0.41), basketball league in Spain (B-Spa, 0.27), and men's world curling championships (C-WC, 0.29).

The difference (ΔWAC_5_) between the observed (aWAC_5_) and the expected (eWAC_5_) WAC_5_ values (ΔWAC_5_ = eWAC_5_ - aWAC_5_) can be used as an indicator of a relative grouping of champions within average 5-year period. ΔWAC_5_ was highly significant (p < 0.01) in 32 competitions, significant (p < 0.05) in nine, suggestive (p < 0.1) in three, and non-significant (p > 0.1) in 24 of them (Table 2). The largest ΔWAC_5_ was observed for women tennis at Australian Open (T-Aus-W, 0.43), followed by several other tennis tournaments (between 0.42 and 0.29) and F1 racing (0.36). These results were expected because individual athletes have shorter active carriers than teams, thus their championship wins have to be grouped. A very high ΔWAC_5_ was found also for F1-E (0.36) because different groups of engines were used at different times in the F1 racing history. The absolute values of ΔWAC_5_ were smaller for golf (0.06–0.17) because both eWAC_5_ and aWAC_5_ were close to the upper tail of the scale, but all of those differences were still either significant (G-US), or highly significant (G-PGA, G-Mas, and G-Open). Among collective sports the largest ΔWAC_5_ of 0.26 was observed for S-Fra, IH-NHL, and R-Ken, followed by 0.25 for S-NCAA. In all of these competitions, the high groupings were likely caused by several ‘dynasties’ winning majority of championships at different time. For example, in France soccer league (S-Fra) AS Saint-Etienne won seven championships in ten years (1967–1976), then Marseille (Olympique de Marseille) won four championships in a row (1989–1992), followed by Lyon (Olympique Lyonnais) winning seven champions in a row between 2002 and 2008, and more recently, Paris Saint-Germain F.C. won six championships in seven years (2013–2019). The competitions with the lowest ΔWAC_5_, indicating no relative grouping of champions as compared to their random distribution was observed for several soccer cup competitions in Slovakia (S-Svk-C, -0.01), UEFA Cup Winners' Cup (S-CWC-C, -0.04), Spain (S-Spa-C, -0.05), and former Czechoslovakia (S-CSR-C, -0.05).

## 7 Discussion

The exact comparison of the results obtained with the indices proposed in this study with previously published findings is not possible because evaluations were performed for different time periods, competitions, and/or using a within-season variance. However, a limited comparison with published studies shows a generally good agreement with the current findings. Analysis of Lorenz curves revealed a smaller competitive balance in B-NBA than in IH-NHL [24]. Both of these leagues exhibited the higher concentration of championships than F-NFL. Both, the aWAC_5_ and eWAC_5_ values for these three competitions have increased in the same order as previously reported: B-NBA (aWAC_5_ = 0.34 and eWAC_5_ = 0.56), IH-NHL (0.44 and 0.71), and F-NFL (0.63 and 0.82) (Table 2). The HHI index has been used to evaluate concentrations of champions over a ten-year period (2006–2016) in eight leagues. Overall, the competitive balance was substantially higher in four North American professional competitions than in top four European soccer leagues [8]. The competitive balance in these competitions decreased in the order: F-NFL, B-NBA, Ba-MLB, IH-NHL, S-Eng, S-Ger, S-Ita (equal to S-Ger), and S-Spa. The current study also revealed a difference between these eight leagues, with the aWAC_5_ values ranging from 0.37 (B-NBA) to 0.69 (Ba-MLB) for the leagues in North America, and from 0.30 (S-Spa) to 0.34 (S-Eng) for the four European soccer leagues, when only the period of 1990–2020 was considered (as these years overlap with those in the previous study). eWAC_5_ values, that were calculated only for the whole evaluated period of 1960–2020, indicated a similar pattern for six out of eight leagues (Table 2). However, the competitive balance in English premiership (S-Eng, eWAC_5_ = 0.62) was estimated to be higher than in B-NBA (eWAC_5_ = 0.56). Results from the present study also agree with the previous findings that Ba-MLB (eWAC_5_ = 0.86) can be considered as the most balanced of the eight leagues and that among the four European soccer leagues S-Eng (eWAC_5_ = 0.62) is the most balanced [38]. However, while previously S-Ita was found to be the most imbalanced league using 1969–2004 data [38], current study shows that the most imbalanced of the four leagues is S-Spa (eWAC_5_ = 0.34), where 46 out of 60 championships (76.7%) were won by only two teams, Real Madrid CF (27 out of 60, 45.0%) and FC Barcelona (19 out of 60, 31.7%). From the soccer leagues analyzed in this current the highest imbalance in concentration of championships was found in S-Sco (aWAC_5_ = 0.19) and S-Por (aWAC_5_ = 0.21). These results match with the earlier report [23].

Several studies evaluated changes in the competitive balance over time, usually in North American leagues and in top European soccer leagues. Feddersen and Maennig [38] examined a within season balance in Ba-MLB, F-NFL, IH-NHL, B-NBA, S-Ita, S-Ger, S-Eng, S-Spa using HHI and several other parameters. For the entire observation period (1969–2004), 12 trends indicated growing imbalance, 19 trends indicated growing balance, and 17 trends were insignificantly different from zero. Jang et al. [12] determined that the balance over time (52–116 evaluated seasons) improved in Ba-MLB, B-NBA, F-NFL, and IH-NHL but decreased in S-Eng, S-Ger, S-Ita, and S-Spa. Based on the aWAC_5_ and *τ* values, the competitive balance significantly increased in IH-NHL, not changed significantly in B-NBA Ba-MLB, F-NFL, and S-Spa, and decreased at the suggestive or significant level in S-Eng (aWAC_5_ only), S-Ita, and S-Ger (Table 3). Considering that these results were detected using a different time period and evaluation parameters, the grouping of leagues is rather similar. Moreover, comparison of data pooled from the top European soccer leagues (Euro-T5) and four of the professional leagues in North America (N.Am-4) shows a growing difference in competitive balance between the two (Figures 9 and 10). While the aWAC_5_ values were similar in the 1960–1989 period (0.46 for Euro-T5, 0.43 for N–Am.-4), they highly significantly increased in N.Am-4 to 0.59 but significantly decreased in Euro-T5 to 0.34 (Table 3). Identical trends were observed using the Mann-Kendall test (*τ* of -0.49 and 0.56 for Euro-T5 and N–Am.-4, respectively). Similarly, the decrease of competitive balance has also been observed on pooled data for cup competitions from these five soccer federations (Euro-T5-C, decrease from 0.70 to 0.61, *τ* = -0.43) (Figures 9 and 10). When a within season competitive balance was evaluated by Groot [39], the author reported increased superiority of champions (1977–2002) in England (S-Eng) and The Netherlands (S-Net), and to the lesser extend also in Italy (S-Ita) and France (S-Fra). Probably the most studied from all soccer leagues was the English Premier League. Szymanski [40] evaluated the number of teams accounting for top positions in 1977–1998 and reported relatively stable competitive balance. Other authors, however, described decline in competitive balance when analyzing the period of 1888–2007 [41], 1947–2004 [42], 1948–2008 [43], 1963–2005 [44] and 1992–2010 [45]. Most of the authors noted that the significant decline in competitive balance started between 1987 to mid-1990s. These data match well with the observations presented in the current study, where aWAC_5_ values decreased from 0.53 in the 1960–1989 period, to 0.34 in the 1990–2020 period. The *τ* value of -0.25 indicated a similar, though nonsignificant trend (Table 3). Though it was not an objective of the current study to identify factors leading to these changes in competitive balance, previous authors suggested that the change may be related to the back-pass rule [46], the Bosman ruling [41], and/or the increased inequalities in resources between clubs [7].

Several indices were previously developed for the evaluation of between-seasons competitive balance [47], including those measuring concentration of championships [25]. The sliding windows approach, described in this study, combined with the stationary block bootstrapping adds to the flexibility of analyses of competitive balance. Depending on specific requirements of the study, the sliding window approach can be used to analyze various indices and lengths of evaluating periods. The minimal length of the sliding window to calculate WAC index is two (WAC_2_), when only two subsequent championships are considered. When the minimal length window is used, the values of WAC_2_ index can only be 0, when a reigning champion defends the title, or 1, when any other team/individual athlete wins the championship. Therefore, this special case of the WAC index can be termed ‘Defending Champion Index’ (DCI) to emphasize that its values depend only on the success of the reigning champion. The average DCI (aDCI = aWAC_2_) value for the whole evaluated period is equal to the proportion (P) of championships that were not won by reigning champions. Though the P and aDCI values are always identical, their confidence intervals are not. For example, if 10 out of 100 championships were won by defending champions, P = aDCI = 0.9. The 95% confidence interval for P goes from 0.83 to 0.95 (range of 0.12) without continuity correction and from 0.82 to 0.95 (range of 0.13) with continuity correction. However, these confidence intervals do not take into consideration the relationship between subsequent DCI values; therefore, they are identical regardless of the spacing between defended championships. In difference, the stationary block bootstrapping approach (with the block geometric mean of 5 and 100,000 bootstrapping) yielded values from 0.75 to 1.00 (range of 0.25) when those ten defended championships were won in a row, but the confidence interval was only from 0.86 to 0.94 (range of 0.08) when those defended championships were spaced evenly (every 10^th^ championship was defended). Thus, the stationary block bootstrapping approach should be used to calculate aDCI confidence intervals even though aDCI = P. Besides aDCI, eDCI and ΔDCI can be calculated for the Defending Champion Index as was previously described for the WAC_5_ index.

## 8 Conclusions

There are fewer measures of between-seasons competitive balance available than for within-season competitive balance. The between seasons competitive balance based on a sliding window approach and the number of champions within a five-year period described in this work thus provides valuable information for sports governing bodies when evaluating the effect of policies (e.g., salary limitations, revenue sharing, draft rules, or roster limits) or other introduced changes (a league or a tournament format, number of teams, etc.). The current study describes indices developed only from the potential or real number of champions thus making them invariant to the total number of teams (if ≥ 5) in the evaluated league (adding weak teams to the league does not increase a between-season competitive balance) or the league/tournament format. The index (WAC_5_) combined with the sliding window approach, however, is flexible enough to indicate gradual changes in the concentration of championships. The indices described in this study are:

- eWAC_5_ that indicates the average, expected concentration on champions at any given 5-year period. The index is based on calculating concentration of championships in a 5-year period from random distribution of actual champions over the whole evaluation period.
- aWAC_5_ that indicates the average, observed concentration of championships at any given 5-year period. The index confidence intervals are determined through block bootstrapping of actual champions and their order over the whole evaluation period. Because this index is based on a short sliding window, it has a good sensitivity to rapid changes in competitive balance.
- ΔWAC_5_ (ΔWAC_5_ = eWAC_5_ - aWAC_5_) that indicates a relative grouping of champions at any given 5-year period as compared to their random distribution determined through permutation.
- DCI that shows the proportion of championships that were not won be reigning champions.

The application of these indices to data collected for 68 competitions and seven datasets with pooled results from multiple competitions revealed:

- A very high competitive balance at all golf tournaments.
- A low competitive balance at several European soccer leagues and ice hockey competitions.
- A substantially larger competitive balance in the domestic soccer cup competition as compared to the league in the same country (federation)
- An increasing competitive balance in several national and international ice hockey competitions.
- A decreasing competitive balance in several European soccer leagues, and domestic cup competitions.
- A growing difference in competitive balance between the most popular North American professional leagues and the top five European soccer leagues.
- A major grouping of champions in sports involving individual athletes, but also some team sport competitions.

## Declarations

### Author contribution statement

Ivan Simko: Conceived and designed the experiments; Performed the experiments; Analyzed and interpreted the data; Wrote the paper.

### Funding statement

This research did not receive any specific grant from funding agencies in the public, commercial, or not-for-profit sectors.

### Data availability statement

Data included in article/supp. material/referenced in article.

### Declaration of interest's statement

The authors declare no conflict of interest.

### Additional information

No additional information is available for this paper.

## Appendix A Supplementary data

Online resource 1 Online resource 1

## References

[1] Queen (1977). News of the World.

[2] Andreff W., Szymanski S. (2006).

[3] Plunkett_Research (2020). https://www.plunkettresearch.com/statistics/Industry-Statistics-Sports-Industry-Statistic-and-Market-Size-Overview/.

[4] SportyCo (2017). https://medium.com/sportyfi/how-big-is-the-sports-industry-630fba219331.

[5] Forrest D., Simmons R. (2002). Outcome uncertainty and attendance demand in sport: the case of English soccer. J. Roy. Stat. Soc.: Series D (The Statistician).

[6] Sanderson A.R., Siegfried J.J. (2003). Thinking about competitive balance. J. Sports Econ..

[7] Penn R., Berridge D. (2019). Competitive balance in the English premier league. European Journal for Sport and Society.

[8] Leeds M.A., Von Allmen P., Matheson V.A. (2018).

[9] Szymanski S. (2003). The economic design of sporting contests. J. Econ. Lit..

[10] Depken C.A. (1999). Free-agency and the competitiveness of major league baseball. Rev. Ind. Organ..

[11] Humphreys B.R. (2002). Alternative measures of competitive balance in sports leagues. J. Sports Econ..

[12] Jang H., Lee Y.H., Fort R. (2019). Winning in professional team sports: historical moments. Econ. Inq..

[13] Koning R.H. (2009). Sport and measurement of competition. Economist.

[14] Owen P.D., King N. (2015). Competitive balance measures in sports leagues: the effects of variation in season length. Econ. Inq..

[15] Owen P.D., Ryan M., Weatherston C.R. (2007). Measuring competitive balance in professional team sports using the herfindahl-hirschman index. Rev. Ind. Organ..

[16] Fort R., Quirk J. (1995). Cross-subsidization, incentives, and outcomes in professional team sports leagues. J. Econ. Lit..

[17] Horowitz I. (1997). The increasing competitive balance in major league baseball. Rev. Ind. Organ..

[18] Koning R.H. (2000). Balance in competition in Dutch soccer. J. Roy. Stat. Soc.: Series D (The Statistician).

[19] Zheng J., Oh T., Kim S., Dickson G., De Bosscher V. (2018). Competitive balance trends in elite table tennis: the olympic games and world championships 1988-2016. J. Sports Sci..

[20] Lopez M.J., Matthews G.J., Baumer B.S. (2018). How often does the best team win? A unified approach to understanding randomness in North American sport. Ann. Appl. Stat..

[21] Scully G.W. (1989).

[22] Noll R.G., Mangan J.A., Staudohar P.D. (1991). The Business of Professional Sports.

[23] Gerrard B., Fort R.D., Fizel J. (2004). International Sports Economics Comparisons.

[24] Quirk J., Fort R.D. (1997).

[25] Eckard E.W. (1998). The NCAA cartel and competitive balance in college football. Rev. Ind. Organ..

[26] Peet R.K. (1975). Relative diversity indices. Ecology.

[27] Simpson E.H. (1949). Measurement of diversity. Nature.

[28] Rhoades S.A. (1993). The Herfindahl-Hirschman index. Fed. Reserv. Bull..

[29] Shannon C.E. (1948). A mathematical theory of communication. Bell System Technical Journal.

[30] Pielou E.C. (1966). The measurement of diversity in different types of biological collections. J. Theor. Biol..

[31] Ceriani L., Verme P. (2012). The origins of the Gini index: extracts from Variabilità e Mutabilità (1912) by Corrado Gini. J. Econ. Inequal..

[32] Kunsch H.R. (1989). The Jackknife and the Bootstrap for general sationary observations. Ann. Stat..

[33] Politis D.N., Romano J.P. (1994). The stationary bootstrap. J. Am. Stat. Assoc..

[34] Canty A., Ripley B. (2020).

[35] R_Core_Team: R (2019). https://www.R-project.org/.

[36] Önöz B., Bayazit M. (2012). Block bootstrap for Mann–Kendall trend test of serially dependent data. Hydrol. Process..

[37] Patakamuri S.K., O'Brien N. (2020).

[38] Feddersen A., Maennig W. (2005).

[39] Groot L.F. (2005). European football: back to the 1950s. https://ssrn.com/abstract=726727.

[40] Szymanski S. (2001). Income inequality, competitive balance and the attractiveness of team sports: some evidence and a natural experiment from English soccer. Econ. J..

[41] Lee Y.H., Fort R. (2012). Competitive balance: time series lessons from the English premier league. Scot. J. Polit. Econ..

[42] Michie J., Oughton C. (2004).

[43] Curran J., Jennings I., Sedgwick J. (2009). ‘Competitive balance’in the top level of English football, 1948–2008: an absent principle and a forgotten ideal. Int. J. Hist. Sport.

[44] Goossens K. (2006). Competitive balance in European football: comparison by adapting measures: national measures of seasonal imbalance and top 30. Rivista di Diritto ed Economia Dello Sport.

[45] Ramchandani G. (2012). Competitiveness of the English premier league (1992-2010) and ten European football leagues (2010). Int. J. Perform. Anal. Sport.

[46] Kent R.A., Caudill S.B., Mixon F.G. (2013). Rules changes and competitive balance in European professional soccer: evidence from an event study approach. Appl. Econ. Lett..

[47] Manasis V., Ntzoufras I. (2014). Between-seasons CompetitiveBalance in European football: review of existing and development of specially designed indices. J. Quant. Anal. Sports.

