# Supplementary material for: We are the champions. The index for evaluating concentration of championships using a sliding window approach
Source: Heliyon. 2022 Dec 5;8(12):e12025. doi: 10.1016/j.heliyon.2022.e12025 (PMC9747587; doi:10.1016/j.heliyon.2022.e12025)
Supplement: Online resource 1 [file mmc1.docx]

Supplemental file 1. Links to original data (April 27, 2020).

| Competition^a^ | Sport | Competition name | Link | Note |
| --- | --- | --- | --- | --- |
| B-NBA | Basketball | National Basketball Association | <https://en.wikipedia.org/wiki/List_of_NBA_champions> |  |
| B-NCAA | Basketball | National Collegiate Athletic Association | <https://en.wikipedia.org/wiki/List_of_NCAA_Division_I_men%27s_basketball_champions> |  |
| B-NCAA-W | Basketball | National Collegiate Athletic Association | <https://en.wikipedia.org/wiki/NCAA_Division_I_Women%27s_Basketball_Tournament> |  |
| B-Spa | Basketball | Liga Endesa | <https://en.wikipedia.org/wiki/Liga_Española_de_Baloncesto_(1957–1983)>  <https://en.wikipedia.org/wiki/Liga_ACB> |  |
| Ba-Jap | Baseball | Japan Championship Series | <https://en.wikipedia.org/wiki/Japan_Series> |  |
| Ba-Mex | Baseball | Mexican Pacific League | <https://en.wikipedia.org/wiki/Mexican_Pacific_League> |  |
| Ba-MLB | Baseball | Major League Baseball | <https://en.wikipedia.org/wiki/List_of_World_Series_champions> |  |
| C-WC | Curling | World Curling Championship | <https://en.wikipedia.org/wiki/World_Curling_Championships> |  |
| C-WC-W | Curling | World Curling Championship | <https://en.wikipedia.org/wiki/World_Curling_Championships> |  |
| F-AFL | Football-AU | Australian Football League | <https://en.wikipedia.org/wiki/List_of_VFL/AFL_premiers> |  |
| F-NFL | Football-US | National Football League | <https://en.wikipedia.org/wiki/List_of_Super_Bowl_champions> |  |
| F1 | Formula 1 | Formula One World Drivers’ Championship | <https://en.wikipedia.org/wiki/List_of_Formula_One_World_Drivers%27_Champions> |  |
| F1-E^a^ | Formula 1 | Formula One Championship - Engine | <https://en.wikipedia.org/wiki/List_of_Formula_One_World_Drivers%27_Champions> | Not an official competition |
| G-Mas | Golf | Masters Tournament | <https://en.wikipedia.org/wiki/Men%27s_major_golf_championships> |  |
| G-Open | Golf | British Open | <https://en.wikipedia.org/wiki/Men%27s_major_golf_championships> |  |
| G-PGA | Golf | PGA Championship | <https://en.wikipedia.org/wiki/Men%27s_major_golf_championships> |  |
| G-US | Golf | U.S. Open | <https://en.wikipedia.org/wiki/Men%27s_major_golf_championships> |  |
| IH-NHL | Ice hockey | National Hockey League | <https://en.wikipedia.org/wiki/List_of_Stanley_Cup_champions> |  |
| IH-Rus | Ice hockey | Kontinental Hockey League | <https://en.wikipedia.org/wiki/List_of_Soviet_and_Russian_ice_hockey_champions> | Combined with USSR |
| IH-Svk | Ice hockey | Slovak Extraliga | <https://en.wikipedia.org/wiki/List_of_Slovak_ice_hockey_champions> |  |
| IH-Swe | Ice hockey | Swedish Hockey League | <https://en.wikipedia.org/wiki/List_of_Swedish_ice_hockey_champions> |  |
| IH-WC | Ice hockey | World Championship & Olympic Games | <https://en.wikipedia.org/wiki/List_of_IIHF_World_Championship_medalists>  <https://en.wikipedia.org/wiki/List_of_Olympic_medalists_in_ice_hockey> | Combined with Olympics winners |
| IH-WC-W | Ice hockey | World Championship & Olympic Games | <https://en.wikipedia.org/wiki/IIHF_World_Women%27s_Championship>  <https://en.wikipedia.org/wiki/List_of_Olympic_medalists_in_ice_hockey> | Combined with Olympics winners |
| R-Eng | Rugby | Premiership Rugby | <https://en.wikipedia.org/wiki/Premiership_Rugby> |  |
| R-Fra | Rugby | French National Rugby League (Top 14) | <https://en.wikipedia.org/wiki/Top_14> |  |
| R-I&NI | Rugby | All-Ireland League | <https://en.wikipedia.org/wiki/All-Ireland_League_(rugby_union)> |  |
| R-Ken | Rugby | Kenya Cup | <https://en.wikipedia.org/wiki/Kenya_Cup> |  |
| S-Bel | Soccer | Belgian Football Championship | <https://en.wikipedia.org/wiki/List_of_Belgian_football_champions> |  |
| S-Bel-C | Soccer | Belgian Cup | <https://en.wikipedia.org/wiki/List_of_Belgian_Cup_finals> |  |
| S-Bra | Soccer | Brazilian Football Championship | <https://en.wikipedia.org/wiki/List_of_Brazilian_football_champions> |  |
| S-CL | Soccer | UEFA Champions League | <https://en.wikipedia.org/wiki/List_of_European_Cup_and_UEFA_Champions_League_finals> |  |
| S-CL-C^a^ | Soccer | UEFA Champions League - Country | <https://en.wikipedia.org/wiki/List_of_European_Cup_and_UEFA_Champions_League_finals> | Not an official competition |
| S-CSR | Soccer | Czechoslovak First League | <https://en.wikipedia.org/wiki/Czechoslovak_First_League> |  |
| S-CSR-C | Soccer | Czechoslovak Cup | <https://en.wikipedia.org/wiki/Czechoslovak_Cup> |  |
| S-CWC | Soccer | UEFA Cup Winners' Cup | <https://en.wikipedia.org/wiki/List_of_UEFA_Cup_Winners%27_Cup_finals> |  |
| S-CWC-C^a^ | Soccer | UEFA Cup Winners' Cup - Country | <https://en.wikipedia.org/wiki/List_of_UEFA_Cup_Winners%27_Cup_finals> | Not an official competition |
| S-Cze | Soccer | Czech Football Championship | <https://en.wikipedia.org/wiki/List_of_Czech_football_champions> | No individual data when S-CSR |
| S-Cze-C | Soccer | Czech Cup | <https://en.wikipedia.org/wiki/Czech_Cup> |  |
| S-EL | Soccer | UEFA Europa League | <https://en.wikipedia.org/wiki/List_of_UEFA_Cup_and_Europa_League_finals> |  |
| S-EL-C^a^ | Soccer | UEFA Europa League - Country | <https://en.wikipedia.org/wiki/List_of_UEFA_Cup_and_Europa_League_finals> | Not an official competition |
| S-Eng | Soccer | Premier League | <http://www.rsssf.com/tablese/engchamp.html> |  |
| S-Eng-C | Soccer | Football Association Cup | <https://en.wikipedia.org/wiki/List_of_FA_Cup_Finals> |  |
| S-Fra | Soccer | French Football Championship | <https://en.wikipedia.org/wiki/List_of_French_football_champions> |  |
| S-Fra-C | Soccer | Coupe de France | <https://en.wikipedia.org/wiki/List_of_Coupe_de_France_finals> |  |
| S-Ger | Soccer | Bundesliga | <https://en.wikipedia.org/wiki/List_of_German_football_champions> | Combined with West Germany |
| S-Ger-C | Soccer | DFB-Pokal | <https://en.wikipedia.org/wiki/DFB-Pokal> | Combined with West Germany |
| S-Ita | Soccer | Serie A | <https://en.wikipedia.org/wiki/List_of_Italian_football_champions> |  |
| S-Ita-C | Soccer | Coppa Italia | <https://en.wikipedia.org/wiki/Coppa_Italia> |  |
| S-MLS | Soccer | Major League Soccer | <https://en.wikipedia.org/wiki/MLS_Cup> |  |
| S-NCAA | Soccer | National Collegiate Athletic Association | <https://en.wikipedia.org/wiki/NCAA_Division_I_Men%27s_Soccer_Tournament> |  |
| S-Net | Soccer | Eredivisie | <https://en.wikipedia.org/wiki/List_of_Dutch_football_champions> |  |
| S-Net-C | Soccer | KNVB Cup | <https://en.wikipedia.org/wiki/KNVB_Cup> |  |
| S-Por | Soccer | Primeira Liga | <https://en.wikipedia.org/wiki/List_of_Portuguese_football_champions> |  |
| S-Por-C | Soccer | Taça de Portugal | <https://en.wikipedia.org/wiki/Taça_de_Portugal> |  |
| S-Sco | Soccer | Premier Division | <http://www.rsssf.com/tabless/scotchamp.html> |  |
| S-Sco-C | Soccer | Scottish Cup | <https://en.wikipedia.org/wiki/List_of_Scottish_Cup_finals> |  |
| S-Spa | Soccer | La Liga | <https://en.wikipedia.org/wiki/List_of_Spanish_football_champions> |  |
| S-Spa-C | Soccer | Copa del Rey | <https://en.wikipedia.org/wiki/Copa_del_Rey> |  |
| S-Svk | Soccer | Slovak Super Liga | <https://en.wikipedia.org/wiki/Slovak_Super_Liga> | No individual data when S-CSR |
| S-Svk-C | Soccer | Slovak Cup | <https://en.wikipedia.org/wiki/Slovak_Cup> |  |
| T-Aus | Tennis | Australian Open | <https://en.wikipedia.org/wiki/List_of_Australian_Open_men%27s_singles_champions> |  |
| T-Aus-W | Tennis | Australian Open | <https://en.wikipedia.org/wiki/List_of_Australian_Open_women%27s_singles_champions> |  |
| T-Eng | Tennis | Wimbledon | <https://en.wikipedia.org/wiki/List_of_Wimbledon_gentlemen%27s_singles_champions> |  |
| T-Eng-W | Tennis | Wimbledon | <https://en.wikipedia.org/wiki/List_of_Wimbledon_ladies%27_singles_champions> |  |
| T-Fra | Tennis | French Open | <https://en.wikipedia.org/wiki/List_of_French_Open_men%27s_singles_champions> |  |
| T-Fra-W | Tennis | French Open | <https://en.wikipedia.org/wiki/List_of_French_Open_women%27s_singles_champions> |  |
| T-USA | Tennis | US Open | <https://en.wikipedia.org/wiki/List_of_US_Open_men%27s_singles_champions> |  |
| T-USA-W | Tennis | US Open | <https://en.wikipedia.org/wiki/List_of_US_Open_women%27s_singles_champions> |  |

^a^ Indicates four competitions that are not official but were added to the list to evaluate additional trends.
